# Nanosecond Pulsed Electric Fields (nsPEFs) for Precision Intracellular Oncotherapy: Recent Advances and Emerging Directions

**DOI:** 10.3390/ijms262311268

**Published:** 2025-11-21

**Authors:** Kainat Gul, Sohail Mumtaz

**Affiliations:** 1 Department of Botany, Hazara University, Mansehra 21120, Pakistan; 2 Department of Chemical and Biological Engineering, Gachon University, 1342 Seongnamdaero, Sujeong-gu, Seongnam-si 13120, Republic of Korea

**Keywords:** nanosecond pulsed electric fields (nsPEFs), intracellular oncotherapy, electroporation cancer therapy, nanoparticle-assisted nsPEF, non-thermal tumor ablation

## Abstract

Intracellular targeting is the missing dimension in contemporary oncology, and nanosecond pulsed electric fields (nsPEFs) uniquely aim to deliver it. By charging membranes on sub-microsecond timescales, nsPEF bypasses plasma-membrane shielding to porate organelles, collapse mitochondrial potential, perturb ER calcium, and transiently open the nuclear envelope. This mechanism reprograms malignant fate while preserving tissue architecture. This review synthesizes the most recent evidence to frame nsPEF as a programmable intracellular therapy, mapping mechanistic design rules that link pulse width, amplitude, repetition, and rise time to specific organelle responses. We outline therapeutic applications, including the induction of apoptosis in resistant tumors, immunogenic cell death with systemic memory, and synergy with checkpoint blockade. We also survey integrations with nanoparticles, calcium, and chemotherapeutic drugs for improved outcomes. We critically appraise safety, selectivity, and scalability, distill translational bottlenecks in dosimetry and standardization, and propose an actionable roadmap to accelerate clinical adoption. Viewed through this lens, nsPEF is not merely another ablation tool but a platform for precision intracellular oncotherapy, capable of drug-sparing efficacy and immune convergence when engineered with rigor.

## 1. Introduction

### 1.1. Overview of nsPEF as a Transformative Oncology Tool

The last two decades have witnessed the emergence of nanosecond pulsed electric fields (nsPEFs) as a transformative modality in bioelectronic oncology [1,2,3,4,5]. nsPEFs are characterized by ultrashort, high-intensity pulses (typically 10–300 nanoseconds, 10–100 kV/cm), which generate unique dielectric responses within living tissues [5]. Unlike conventional microsecond or millisecond electroporation, nsPEFs operate in the subcellular frequency domain, allowing electric fields to penetrate beyond the plasma membrane and induce ultrafast polarization of intracellular structures [5,6]. This capacity to directly target organelles such as mitochondria, endoplasmic reticulum (ER), and nucleus without relying on exogenous agents sets nsPEF apart as a non-thermal, non-chemical, and highly versatile oncotherapy tool [7,8].

Preclinical studies have demonstrated the striking ability of nsPEFs to selectively ablate solid tumors, suppress metastatic spread, and prime systemic anti-tumor immunity while sparing adjacent normal tissue [7,9,10,11,12,13,14,15]. This dual action, direct cytotoxicity and immune activation mirrors the hallmarks of successful oncotherapies, but is achieved through purely physical bioelectric mechanisms [16]. Moreover, nsPEFs circumvent several limitations of traditional therapies: they are not constrained by drug solubility or bioavailability, not subject to pharmacokinetic clearance, and not reliant on genetic targeting that may be undermined by tumor heterogeneity [16]. In this sense, nsPEFs are not merely incremental improvements over existing modalities; instead, they represent a paradigm shift in how energy-based medicine can be harnessed for intracellular oncotherapy [5].

**Figure 1:**
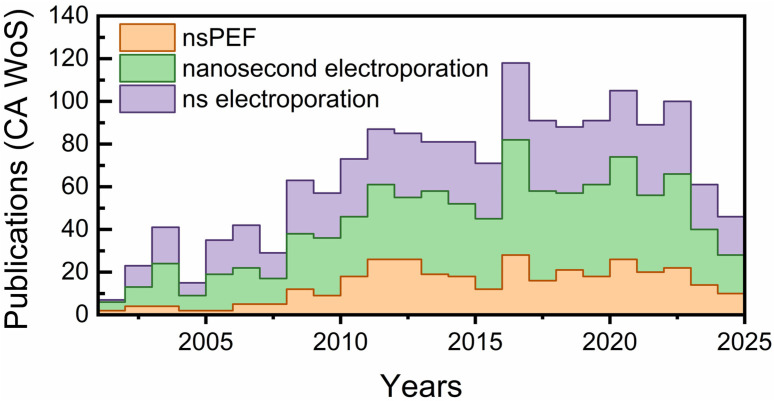
Trends in global publications on nsPEFs and related technologies over the past two decades. The number of articles indexed in CA Web of Science using the terms “nsPEF,” “nanosecond electroporation,” and “ns electroporation”.

The publication landscape over the last two decades underscores the rapid evolution of nsPEFs from a niche bioelectrical phenomenon to a recognized tool in oncology and biomedical engineering. As shown in Figure 1, research output steadily increased after 2005, with notable surges corresponding to breakthroughs in mechanistic modeling, intracellular targeting, and preclinical applications. The parallel rise in terms such as “nanosecond electroporation” reflects the field’s expansion into diverse contexts, including drug delivery, immunotherapy, and precision ablation. Interestingly, the apparent stabilization in publication rate after 2020 may reflect multiple factors, including global research disruptions, funding realignments, and the growing complexity of clinical and translational investigations, rather than a simple indicator of field maturity. This trajectory reinforces the timeliness of a comprehensive review on nsPEFs, highlighting both the progress made and the remaining challenges to establish the technology as a mainstream modality in precision intracellular oncotherapy.

### 1.2. Significance of Intracellular Targeting in Cancer Therapy

Cancer biology has long emphasized the centrality of intracellular processes, apoptosis resistance, metabolic rewiring, DNA repair, and immune evasion in determining therapeutic outcomes [17,18,19,20]. Most chemotherapies, targeted agents, and even immunotherapies ultimately converge on modulating these intracellular nodes. However, pharmacological strategies face formidable barriers: (i) limited drug penetration into tumor cores; (ii) multidrug resistance via efflux pumps or enzymatic inactivation; (iii) mutations in drug-binding sites; (iv) dose-limiting systemic toxicities. Preclinical investigations have demonstrated that nsPEFs significantly reduce CSC-associated subpopulations, including CD44^+^/CD24^−^ cells in breast cancer xenografts and CD133^+^ glioma stem-like cells, resulting in diminished tumor-initiating capacity and delayed recurrence in vivo [21,22,23,24]. These findings suggest that nsPEFs can selectively disrupt stem-like niches that drive therapeutic resistance and relapse.

Here lies the significance of nsPEFs: they provide a physical means of intracellular targeting that bypasses these biochemical resistance mechanisms [19,25,26,27]. By permeabilizing internal membranes, nsPEFs release Ca^2+^ from the ER, disrupt mitochondrial membrane potential, induce reactive oxygen species (ROS) generation, and perturb nuclear chromatin structure within nanoseconds [8,28,29,30,31,32]. These effects converge on apoptosis and immunogenic cell death (ICD), both of which are highly desirable in precision oncology [33]. Unlike necrosis, ICD involves the exposure of calreticulin, release of adenosine triphosphate (ATP), and secretion of HMGB1—danger-associated molecular patterns (DAMPs) that stimulate dendritic cells and activate adaptive immune responses [33,34]. Thus, nsPEFs not only eliminate local tumor cells but also convert the tumor into an in situ vaccine, amplifying their therapeutic relevance in the era of immunotherapy [35,36,37].

A second dimension of significance is cancer stem cell (CSC) targeting [38]. CSCs represent a small subpopulation within tumors responsible for relapse and metastasis due to their resistance to conventional chemotherapy and radiotherapy [39,40]. Early evidence suggests that nsPEFs can compromise CSC viability by disrupting their mitochondrial bioenergetics and self-renewal capacity, thereby addressing one of the most intractable barriers in oncology [21,22,23,24]. Collectively, these attributes make nsPEFs uniquely suited to meet the pressing demands of modern oncology: therapies that are precise, minimally invasive, resistance-proof, and capable of synergizing with immunotherapeutic platforms

### 1.3. A Broader Context: From Electroporation to Precision Bioelectric Medicine

**Figure 2:**
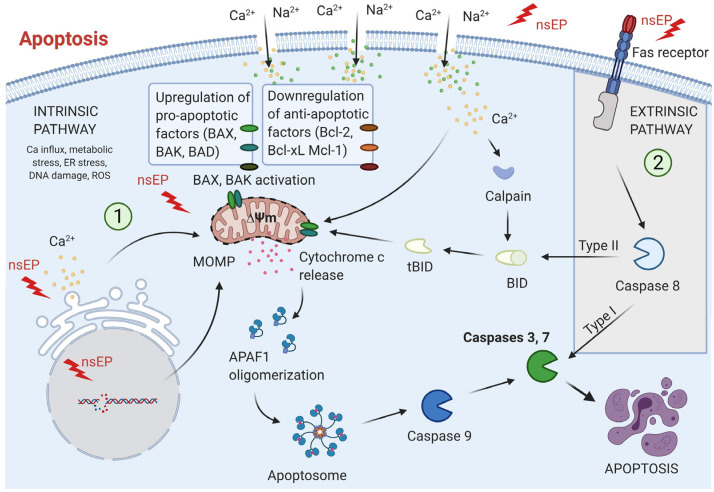
Apoptotic pathways induced by nanosecond pulse electroporation (nsEP): Apoptosis triggered by nsEP primarily follows the intrinsic (mitochondrial) pathway, initiated by factors such as intracellular Ca^2+^ elevation, oxidative and metabolic stress, ER disruption, DNA damage, and ROS generation. These signals promote pro-apoptotic proteins, suppress anti-apoptotic regulators, collapse mitochondrial membrane potential, and induce mitochondrial outer membrane permeabilization (MOMP). Subsequent release of cytochrome c enables apoptosome assembly, caspase-9 activation, and downstream activation of caspases-3/7, culminating in cell death. In certain cell types, nsEP can also activate the extrinsic pathway, where Fas receptor clustering stimulates caspase-8. This either directly activates caspases-3/7 (Type I) or engages the mitochondrial pathway for amplification (Type II). Adopted from [41].

Electroporation has a long history in biomedical science, from enhancing gene transfer in basic research to enabling electrochemotherapy in clinical oncology [41,42,43,44]. However, traditional approaches using microsecond or millisecond pulses are largely restricted to transiently permeabilizing the plasma membrane for drug delivery. nsPEFs expand this paradigm by shifting the electroporation target inward [1,41,45,46]. Their ultrashort duration allows them to bypass membrane charging times and distribute across intracellular compartments with sub-nanosecond precision (Figure 2).

This positions nsPEFs within the broader field of precision bioelectric medicine, a discipline that views electrical energy not only as a means of tissue ablation, but as a programmable tool to modulate cellular fate. The integration of nsPEFs with advanced imaging, nanomedicine, and AI-driven pulse design further elevates their relevance. For example, nanoparticle-assisted nsPEF delivery can enhance tumor specificity, while machine learning can optimize pulse combinations for individual tumor phenotypes, thereby achieving true personalization [41,47]. In this way, nsPEFs embody the convergence of physics, engineering, and oncology [41,48,49,50].

### 1.4. Objectives of the Review

The central objective of this review is to provide a comprehensive and forward-looking synthesis of nsPEFs in the context of precision intracellular oncotherapy. Specifically, we aim to dissect the fundamental biophysical mechanisms by which nsPEFs permeabilize intracellular membranes and reprogram the fate of cancer cells, with particular emphasis on organelle-specific responses such as mitochondrial depolarization, endoplasmic reticulum calcium release, and nuclear perturbations. Beyond mechanistic insight, this review critically evaluates the translational applications of nsPEFs, including their capacity to induce apoptosis in resistant tumor cells, promote immunogenic cell death, and eradicate cancer stem cell populations that drive relapse and metastasis. Finally, we highlight innovative frontiers that may define the future trajectory of this technology, such as nanoparticle-mediated precision delivery, integration with immunotherapy and gene-editing platforms, real-time imaging for treatment monitoring, and artificial intelligence–driven optimization of pulse protocols for patient-specific therapy. By uniting mechanistic depth with translational scope, this review seeks to position nsPEFs not as an experimental curiosity, but as a clinically actionable modality capable of reshaping the paradigm of intracellular oncology.

## 2. Intracellular Electrophysiology of nsPEF

### 2.1. Ultrafast Electroporation and Membrane Charging

The defining feature of nsPEFs lies in their ability to induce ultrafast electroporation through dielectric charging of cellular and subcellular membranes within nanoseconds [51,52,53]. In contrast to conventional microsecond pulses, where the electric field primarily accumulates across the plasma membrane due to its relatively long charging constant (on the order of microseconds), the nanosecond duration of nsPEFs circumvents this temporal bottleneck [53]. As a result, the electric field penetrates deep into the cytoplasm, distributing across intracellular structures including mitochondria, endoplasmic reticulum, and nuclear envelope. Electroporation is fundamentally governed by the Schwan equation, which predicts the transmembrane potential (Δ*V_m_*) induced by an external field [54]:

Δ*V_m_* = 1.5·*E*·*r*·*cosθ* (1)

where *E* is the applied field, *r* is the cell radius, and *θ* is the polar angle. For mammalian cells (10–20 μm in diameter), field strengths of 10–20 kV/cm applied over 100–300 ns can generate Δ*V_m_* values sufficient to surpass the critical threshold (0.2–1 V) required for electropore formation [55]. Importantly, because the pulse duration is shorter than the charging time of the plasma membrane (1 μs), nsPEFs prevent exclusive localization of the field at the outer membrane, enabling simultaneous charging of internal organelles with smaller characteristic sizes and faster time constants [56].

The pores generated by nsPEFs are distinct from those formed under longer pulse conditions [57]. Instead of large, stable nanopores, nsPEFs typically create transient, nanoscale defects with lifetimes of milliseconds to seconds [57]. These nanopores are sufficient to allow selective passage of small ions such as Ca^2+^ while restricting macromolecules, thereby modulating signaling without catastrophic membrane rupture. Such ultrafast electroporation therefore serves as a precision tool, perturbing bioelectric gradients while preserving global membrane integrity [58]. A further distinguishing property is the distributed charging across multiple dielectric barriers. Organelles with thin lipid bilayers (5 nm) and smaller capacitances reach the electroporation threshold more rapidly than the plasma membrane. This explains why nsPEFs trigger mitochondrial depolarization, ER calcium release, and nuclear perturbations within seconds of pulse delivery [59]. Thus, rather than being restricted to a single site of entry, nsPEFs orchestrate a multi-organelle bioelectric perturbation, uniquely positioning them to reprogram cancer cell physiology at its intracellular core.

From a therapeutic perspective, this property holds immense promise [60,61,62,63]. Conventional electroporation is largely restricted to drug delivery, where the plasma membrane is a gatekeeper. In contrast, nsPEFs act as direct modulators of intracellular electrophysiology, altering ion homeostasis, bioenergetic flux, and redox signaling with nanosecond precision. This capability not only distinguishes nsPEFs from other ablation methods such as radiofrequency or thermal therapies but also provides a foundation for synergistic oncotherapeutic strategies where intracellular vulnerabilities are exploited without collateral tissue damage [62,64,65].

### 2.2. Organelle-Specific Responses: Mitochondria, Endoplasmic Reticulum, and Nucleus

One of the most distinctive hallmarks of nsPEF therapy is its ability to extend electroporation beyond the plasma membrane and perturb the physiology of intracellular organelles and mitochondrial outer membrane permeabilization (MOMP). This sets nsPEFs apart from longer-pulse modalities, whose effects remain largely confined to the plasma membrane. By exploiting nanosecond-scale dielectric relaxation, nsPEFs induce multi-organelle bioelectric perturbations that reshape signaling pathways central to cancer cell survival and immune evasion [66,67].

#### 2.2.1. Mitochondria

Mitochondria are particularly sensitive to nsPEFs due to their small size, high surface-to-volume ratio, and short membrane charging constants [68,69]. Exposure to nsPEFs results in rapid depolarization of the mitochondrial membrane potential (ΔΨm), accompanied by swelling, cristae disorganization, and increased production of ROS [70]. Recent work in cardiomyocytes and cancer models has demonstrated that 100 ns pulses trigger outer mitochondrial membrane (OMM) permeabilization without immediately collapsing the inner membrane, leading to cytochrome-c release and initiation of intrinsic apoptosis [71]. Additional studies have revealed that mitochondrial susceptibility is partially regulated by cyclophilin D, suggesting a convergence between nsPEF-induced pores and the mitochondrial permeability transition pore complex [72,73,74,75]. These findings position mitochondria as a central execution hub in nsPEF-mediated cell death, and importantly, highlight that selective targeting of mitochondrial bioenergetics could underlie the specificity of nsPEFs toward malignant cells with heightened metabolic stress [68,76,77].

#### 2.2.2. Endoplasmic Reticulum (ER)

The ER, as the principal calcium reservoir of the cell, is also a primary nsPEF target [78,79,80]. The nsPEFs induce acute calcium efflux from the ER lumen, disrupting cytosolic Ca^2+^ homeostasis within milliseconds. This rapid surge triggers ER stress pathways, activates unfolded protein response (UPR) signaling, and promotes cross-talk with mitochondria through mitochondria-associated membranes (MAMs) [81]. Research provided direct evidence that nsPEFs alter ER morphology and cytoskeletal integrity in cancer cells, correlating ER disruption with mitochondrial depolarization and apoptotic priming. The release of ER Ca^2+^ further amplifies ROS production and contributes to the induction of ICD, making the ER not only a mechanistic driver of apoptosis but also a critical immunomodulatory organelle in the nsPEF response cascade [82,83,84,85].

#### 2.2.3. Nucleus

Although the nuclear envelope shields nuclear DNA, nsPEFs exert direct and indirect effects on nuclear dynamics [86]. On the one hand, dielectric charging of the nuclear envelope can produce transient pores, enabling ion flux and perturbing nucleocytoplasmic transport [87]. On the other hand, secondary signaling from mitochondrial ROS and ER stress leads to chromatin condensation, DNA fragmentation, and modulation of repair pathways. Yao et al. [80], demonstrated that 5 ns pulses alter nuclear morphology and chromatin architecture in aggressive breast cancer cells, suggesting that nsPEFs may modulate genome stability. Such nuclear perturbations may sensitize tumor cells to DNA-damaging chemotherapies or radiotherapy, offering a combinatorial avenue for clinical translation [88,89,90,91].

Taken together, nsPEFs impose a multi-organelle stress phenotype characterized by mitochondrial depolarization, ER calcium efflux, and nuclear reorganization [92]. This coordinated disruption acts as a fail-safe against tumor resistance: even if one pathway (e.g., mitochondrial apoptosis) is impaired, parallel routes such as ER stress or nuclear damage reinforce cell death signaling. The ability of nsPEFs to simultaneously engage multiple intracellular compartments explains their broad efficacy against drug-resistant tumors and their synergy with immunotherapies. Importantly, these effects arise without exogenous agents, underscoring nsPEFs as a purely physical yet highly selective modality for intracellular oncotherapy [93,94,95,96].

### 2.3. Pulse Parameters for Selective Treatments

The therapeutic efficacy of nsPEFs depends critically on the precise tuning of pulse parameters, which determine whether cells undergo reversible perturbation, regulated cell death, or catastrophic necrosis [97]. Unlike conventional electroporation, which is optimized for transient plasma membrane permeabilization, nsPEFs exploit a multidimensional parameter space, pulse duration, amplitude, repetition rate, number of pulses, and frequency spectrum, to achieve selective intracellular disruption in malignant cells while sparing normal tissues [35].

Cross-study synthesis. Across hepatocellular, lung, and pancreatic models, a consistent boundary emerges between apoptosis-dominant and necrosis-dominant responses as a joint function of pulse width (100–300 ns), field amplitude (≈10–25 kV/cm), and pulse number. Moderate fields with 100–200 ns pulses reproducibly favor mitochondrial depolarization and apoptosis with ICD features, whereas escalation beyond ~25–30 kV/cm or very high cumulative pulse counts increases primary necrosis [35]. This trend holds despite differences in cell line bioenergetics and 2D vs. 3D contexts, suggesting that effective “apoptosis windows” are transferable if local conductivity and electrode geometry are accounted for. Apparent discrepancies in the literature align with unreported differences in rise time and repetition frequency, which influence how effectively the plasma-membrane capacitor is bypassed; standardizing these reporting elements reduces the perceived conflict and clarifies dose–response rules.

#### 2.3.1. Pulse Duration (Width)

Pulse width dictates how electric fields interact with cellular membranes and intracellular organelles [40]. Ultrashort pulses (1–10 ns) have rise times fast enough to overcome the plasma membrane charging delay, allowing the field to penetrate into the cytoplasm and directly polarize organelles. Recent simulations and experimental validation in MDA-MB-231 breast cancer cells confirmed that 5 ns pulses can disrupt the endoplasmic reticulum (ER), mitochondria, and nuclear envelope without catastrophic plasma membrane rupture, highlighting their unique role in organelle-specific targeting [97].

By contrast, longer ns pulses (100–300 ns) generate sustained plasma membrane charging, resulting in robust Ca^2+^ influx, osmotic imbalance, and apoptotic priming. In hepatocellular carcinoma and melanoma models, 100 ns pulses triggered mitochondrial depolarization, caspase activation, and effective tumor shrinkage with minimal necrosis [98,99,100]. The distinction suggests that pulse width functions as a “therapeutic dial”: ultrashort pulses favor precision modulation of organelles, while longer ns pulses favor bulk tumor ablation [101].

#### 2.3.2. Field Strength (Amplitude)

Amplitude determines the magnitude of the induced transmembrane potential (ΔV_m) across both plasma and organelle membranes. A critical threshold of 10–20 kV/cm is generally required to initiate pore formation in malignant cells, with higher amplitudes (>30–40 kV/cm) producing more extensive permeabilization [100]. Importantly, cancer cells often exhibit lower electroporation thresholds than non-malignant counterparts due to altered membrane composition, resting potential, and dielectric properties. This provides a degree of bioelectric selectivity, enabling malignant cells to be preferentially ablated at field strengths that healthy cells can tolerate. Amplitude also dictates the mode of cell death [102,103]. Moderate fields induce apoptosis and ICD, desirable outcomes for precision oncology, whereas excessive amplitudes increase the likelihood of necrosis and collateral tissue damage. Careful amplitude control is therefore central to balancing tumor eradication with preservation of tissue architecture and minimizing post-treatment inflammation [104,105].

#### 2.3.3. Pulse Number and Repetition Rate

The number of pulses delivered during treatment defines the cumulative energy dose and thus the biological endpoint. Low pulse counts (<100) frequently produce reversible stress responses, such as transient mitochondrial depolarization or ER Ca^2+^ release, without committing cells to apoptosis. In contrast, higher pulse counts (500–1000) lead to irreversible apoptosis, caspase activation, and release of DAMPs that initiate ICD [80,106]. Repetition rate further modulates treatment outcome. At low frequencies (1–10 Hz), cells have time to partially recover between pulses, favoring regulated apoptosis pathways. At high frequencies (>1 kHz), recovery is limited, cumulative thermal effects can emerge, and necrosis becomes more likely [107,108,109]. This dimension allows nsPEFs to be tuned either toward controlled apoptosis with immune synergy or toward rapid ablation, depending on clinical context.

#### 2.3.4. Frequency Spectrum and Rise Time

A distinctive feature of nsPEFs is their broadband frequency content in the MHz–GHz range, resulting from their ultrafast rise times (<5 ns). These high-frequency components enable penetration of the plasma membrane’s capacitive shield, allowing direct organelle polarization. Computational analyses and AI-driven modeling indicate that faster rise times (<3 ns) maximize energy delivery into mitochondria and nucleus, while slower rise times (>10 ns) limit effects primarily to the plasma membrane [110]. This spectral property explains why nsPEFs are uniquely capable of targeting multiple organelles. Adjusting rise time and frequency content provides a means of biasing the therapy toward specific intracellular compartments, an emerging frontier in personalized pulse design [111,112].

#### 2.3.5. Tumor Selectivity and Microenvironmental Influences

Malignant cells display unique dielectric signatures compared to normal tissues, including higher membrane potential, altered lipid composition, and metabolic stress [113]. These features lower their electroporation thresholds and make them disproportionately susceptible to nsPEFs. Recent preclinical studies demonstrate that hepatocellular carcinoma and breast cancer xenografts are efficiently ablated at pulse regimens that cause negligible structural injury to adjacent stromal tissue [113,114]. Beyond intrinsic cellular differences, the tumor microenvironment (TME) influences parameter sensitivity. Hypoxic and acidic niches can alter ion distribution, while fibrotic tissue increases field attenuation. Adjusting amplitude and pulse number is therefore essential to overcome TME heterogeneity [115]. Selectivity also extends to vascular effects: endothelial cells are relatively resistant to nsPEFs, permitting localized ablation of tumor parenchyma while preserving perfusion pathways [116].

Pulse parameters collectively define the therapeutic identity of nsPEFs. Width determines whether organelles or plasma membranes dominate the response; amplitude sets apoptotic versus necrotic outcomes; pulse number and repetition rate govern dose-dependent progression from reversible stress to ICD; frequency spectrum biases field penetration toward intracellular structures; and tumor selectivity arises from intrinsic bioelectric vulnerabilities [117]. Harnessing these variables not only enhances tumor eradication but also positions nsPEFs as a programmable oncotherapy platform [117,118]. Future translation will depend on the development of parameter libraries for different tumor types, integration of real-time biosensing for intraoperative feedback, and AI-driven pulse optimization for patient-specific therapy. Such advances will ensure that nsPEFs progress from experimental models to scalable, clinically actionable tools for precision intracellular oncotherapy [119].

Pulse parameters act in concert to define intracellular outcomes rather than functioning independently. Very rapid rise times (<5 ns) combined with short pulse durations (≤10 ns) and high field amplitudes (>20 kV/cm) allow the pulse to penetrate the plasma membrane before full capacitive charging, leading to direct perturbation of subcellular membranes, including the ER and nuclear envelope. In contrast, slower rise times and longer pulse widths (≥100 ns) promote plasma-membrane poration first, with secondary effects on mitochondria and intracellular organelles as the field dissipates. Thus, organelle selectivity arises from the interplay of field strength, pulse kinetics, and cellular charging time constants. As shown by Yao et al. (2023) [80], exposure to 5 ns pulses (25 kV/cm, 300 pulses) induced measurable alterations in nuclear morphology and chromatin condensation in tumor cells, confirming that ultrashort pulses can directly influence nuclear architecture through subnanosecond charging dynamics

## 3. Therapeutic Applications of nsPEF in Precision Oncology

The nsPEFs translate biophysical control into therapeutic effect by coupling local tumor ablation with intracellular and immunological sequelae. Across hepatocellular, colorectal, melanoma, and lung models, parameter sets in the 100–300 ns range at moderate fields (≈10–20 kV/cm) reproducibly favor apoptosis with features of immunogenic cell death (ICD), while higher fields or very large pulse numbers increase primary necrosis. Importantly, several studies now link these local effects to systemic immunity—demonstrating dendritic cell activation, expansion of tumor-specific CD8^+^ T cells, and, in select settings, abscopal tumor control—thereby positioning nsPEFs as more than a local cytotoxic modality.

The ultimate clinical value of nsPEFs lies in their translation from a purely physical perturbation to a therapeutic modality capable of selectively eliminating tumors, reprogramming the tumor microenvironment, and synergizing with immunotherapy platforms [119]. Recent evidence suggests that nsPEFs achieve their therapeutic effects through three interconnected pathways: induction of apoptosis in resistant tumors, activation of immunogenic cell death that enhances tumor clearance, and synergy with immune checkpoint inhibitors to amplify systemic antitumor immunity [120,121]. Together, these applications establish nsPEFs as a versatile and programmable component of precision oncology [120,122,123].

Concordance and contradictions. Studies reporting checkpoint modulation and myeloid reprogramming after nsPEF converge on a common theme, antigen exposure with pro-inflammatory cytokine release, yet differ in the magnitude and durability of systemic responses. We attribute this to (i) tumor immunogenicity at baseline, (ii) pulse timing relative to immune-active therapies, and (iii) extent of necrosis, which can blunt antigen presentation. Importantly, models that maintain an apoptosis-skewed regime (moderate fields, controlled pulse numbers) show the most consistent CD8^+^ priming and memory, implying that biophysical tuning is an immunologic dial, not merely an ablative setting.

ICD after nsPEF is characterized by surface exposure of calreticulin, extracellular ATP release, and HMGB1 emission, which together enhance antigen uptake and cross-presentation. In immunocompetent models, these signals are followed by increased intratumoral dendritic cells and expansion of effector/memory CD8^+^ T cells, with concomitant reductions in distant, untreated lesions—an abscopal-type response [16]. Human melanoma cell data show nsPEF-induced modulation of checkpoint pathways (e.g., PD-1, MHC-II) and cytokines consistent with T-cell recruitment [124], while clinical–preclinical studies in liver cancer link durable local control to myeloid reprogramming and CD8^+^ memory formation [125,126]. Together, these findings indicate that nsPEFs can function as an in situ vaccine when parameterized to favor apoptosis/ICD over necrosis.

One of the most compelling features of nsPEFs is their ability to induce apoptosis even in tumors that exhibit resistance to conventional chemotherapies and radiotherapies [127]. Many solid tumors evade apoptosis by altering mitochondrial pathways, overexpressing anti-apoptotic proteins such as Bcl-2, or upregulating DNA repair mechanisms. nsPEFs bypass these biochemical roadblocks by physically permeabilizing organelle membranes and destabilizing bioenergetic homeostasis. Recent studies in hepatocellular carcinoma and breast cancer cells demonstrate that 100–300 ns pulses effectively collapse mitochondrial membrane potential, promote cytochrome-c release, and activate caspase cascades, leading to apoptosis in models previously refractory to chemotherapy [128,129]. This unique ability to directly engage apoptosis machinery without relying on receptor-ligand signaling pathways situates nsPEFs as a promising strategy for overcoming intrinsic and acquired resistance. Furthermore, because nsPEF-induced pores are transient and nanoscale, they often avoid catastrophic necrosis, enabling regulated cell death that maintains tissue integrity while ensuring tumor eradication [130].

In addition to intrinsic tumor cell death, nsPEFs trigger synergistic immunomodulation that enhances clearance of residual disease. Unlike necrosis, which primarily provokes sterile inflammation, nsPEF-induced apoptosis often manifests features of ICD. Hallmarks of ICD, calreticulin exposure, ATP release, and HMGB1 secretion, have been consistently observed following nsPEF treatment, resulting in robust recruitment of dendritic cells and activation of tumor-specific T cells [131]. In murine melanoma and pancreatic cancer models, local nsPEF ablation not only reduced tumor burden at the treated site but also delayed or prevented the growth of distant, untreated tumors, indicating the generation of systemic immune memory [132]. This phenomenon effectively converts the tumor into an in situ vaccine, a property rarely achieved with physical ablation techniques such as radiofrequency or cryoablation. Such immunomodulatory potential is highly relevant for precision oncology, where long-term disease control increasingly depends on engaging both innate and adaptive immune responses.

Perhaps most transformative is the ability of nsPEFs to synergize with immune checkpoint inhibitors (ICIs), a class of therapies that has revolutionized cancer treatment but remains effective in only a subset of patients. Checkpoint inhibitors such as anti-PD-1 and anti-CTLA-4 antibodies rely on pre-existing antitumor immunity, which many “cold” tumors lack [133,134,135]. By inducing ICD and mobilizing immune infiltration, nsPEFs can “heat up” these tumors, rendering them more responsive to checkpoint blockade. Recent preclinical data show that combining nsPEFs with PD-1 blockade significantly improves survival in murine models of triple-negative breast cancer and hepatocellular carcinoma [136,137,138,139]. The combination enhances cytotoxic T-cell infiltration, reduces regulatory T-cell populations, and establishes durable immune memory capable of rejecting rechallenge with tumor cells. Such synergy provides a powerful rationale for integrating nsPEFs into modern immuno-oncology protocols, particularly for patients who fail to respond to checkpoint blockade alone [140,141,142]. Taken together, these therapeutic applications highlight the multifunctionality of nsPEFs: they act directly on cancer cells to overcome resistance, reshape the tumor microenvironment to promote immune clearance, and serve as an immune adjuvant when combined with checkpoint inhibitors. Importantly, these outcomes emerge without reliance on exogenous drugs or genetic modification, reinforcing nsPEFs as a drug-free yet precision-driven oncotherapy platform. The clinical trial enrolled fifteen patients with unresectable primary liver tumors (Barcelona Clinic Liver Cancer stage B–C) who were ineligible for thermal ablation or systemic therapy. Notably, no severe hepatic or systemic complications were observed during the extended follow-up period (>18 months), confirming that nsPEF ablation achieved high local control with durable safety in high-risk cohorts. The growing body of preclinical evidence suggests that the future of nsPEF therapy will not be defined by monotherapy alone, but by its ability to serve as a synergistic hub linking apoptosis induction, immunotherapy, and precision medicine. Because nsPEF exposure can simultaneously upregulate PD-1/PD-L1 while releasing danger signals, immune activation may be tempered by compensatory checkpoint engagement. In this context, ICIs are not merely additive but often necessary to consolidate T-cell effector function and durability. Emerging data suggest that nsPEF-first sequencing, followed by ICI, maximizes antigen release and dendritic priming before checkpoint blockade [124].

**Figure 3:**
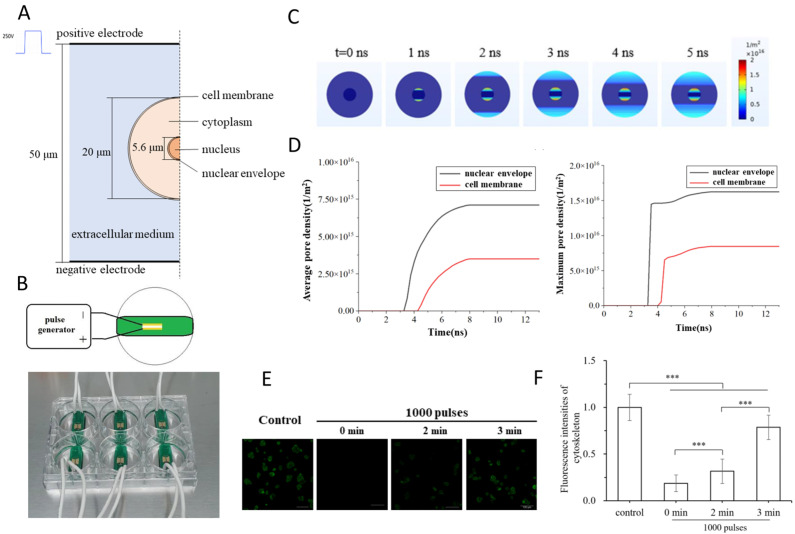
nsPEF-induced electroporation of nuclear and plasma membranes. (**A**) Model of a single cell with a nucleus positioned between electrodes. (**B**) Experimental setup with a custom nsPEF generator and culture chamber. (**C**) Simulation of electric field distribution at 0–5 ns, showing preferential charging of the nuclear envelope. (**D**) Quantification of pore density over time, with nuclear envelope poration exceeding that of the plasma membrane. (**E**) Experimental imaging of propidium iodide uptake after 1000 pulses at different time points. (**F**) Quantitative fluorescence analysis confirming progressive nuclear permeability. Adapted from [80]. (Differences between groups are denoted by *** *p* < 0.001).

Recently, Yao et al. (2023) reported that ultrashort nsPEFs can directly disrupt nuclear structures, a finding of particular relevance to intracellular oncotherapy [80]. In their study, a schematic model of a single cell placed between electrodes (Figure 3A) and the custom-built pulse generator system (Figure 3B) were used to simulate and experimentally validate the effects of 5 ns pulses [80]. The simulation results revealed that the electric field distribution rapidly penetrated the plasma membrane and concentrated on the nuclear envelope within 1–5 ns (Figure 3C), leading to significantly higher pore density in the nuclear envelope compared with the cell membrane (Figure 3D). This mechanistic prediction was confirmed experimentally: propidium iodide fluorescence, monitored over time, demonstrated progressive nuclear uptake following 1000 pulses, with fluorescence intensity increasing markedly from 0 to 3 min (Figure 3E,F). Together, these findings highlight that nsPEFs can bypass the plasma membrane barrier and induce preferential nuclear electroporation, offering a powerful strategy to trigger apoptosis through organelle-specific targeting [80]. While this study establishes a compelling proof of concept, it does not fully address the downstream biological consequences of nuclear envelope permeabilization, including genomic instability, DNA repair signaling, and chromatin remodeling. From an oncological perspective, these outcomes represent both opportunities and challenges: on the one hand, nuclear targeting could enhance the efficacy of gene-editing or nuclear-targeted drug delivery; on the other, genotoxic risks in surrounding normal tissues remain a critical concern. Moving forward, studies should investigate whether nsPEF-mediated nuclear disruption synergizes with DNA-damaging therapies, optimize pulse protocols to minimize off-target toxicity, and extend validation to in vivo tumor models to establish safety and translational potential [143,144].

**Figure 4:**
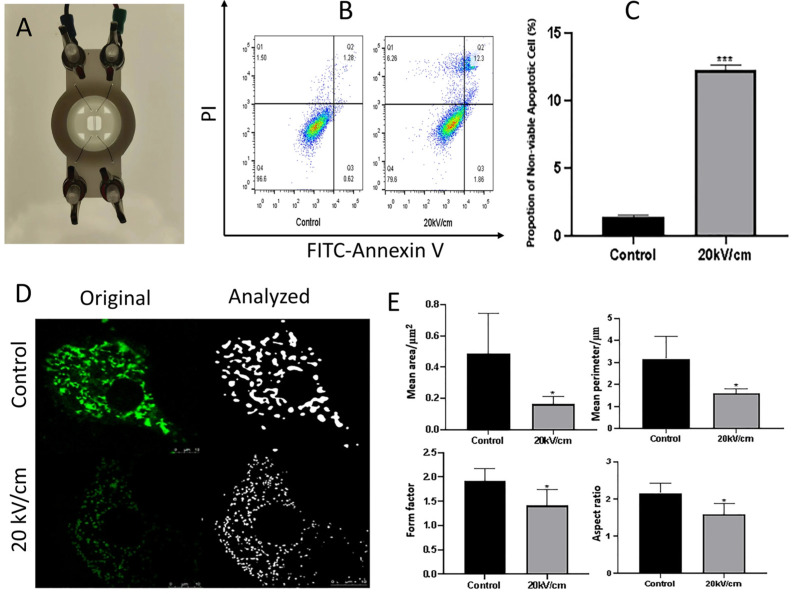
nsPEF-induced mitochondrial apoptosis in rat cardiomyocytes. (**A**) Custom-built electrode setup for cell treatment. (**B**) Flow cytometry dot plots showing Annexin V-FITC/PI staining in control and nsPEF-treated cells. (**C**) Quantification of apoptotic cells, with a significant increase after 20 kV/cm exposure. (**D**) Confocal fluorescence microscopy and image analysis showing mitochondrial fragmentation and structural changes. (**E**) Quantitative measurements revealing decreased mean area, perimeter, form factor, and aspect ratio of mitochondria following nsPEF treatment. Adapted from [145]. (Differences between groups are denoted by * *p* < 0.05, and *** *p* < 0.001).

Fan et al. (2024) investigated how nsPEFs trigger apoptosis through mitochondrial disruption in myocardial cells, offering critical insights into organelle-level responses [145]. In their experimental design, a custom pulse generator and electrode system were employed to deliver 100 ns pulses at 20 kV/cm to rat H9C2 cardiomyocytes (Figure 4A). Flow cytometry analysis using Annexin V-FITC/PI staining demonstrated a marked increase in apoptotic populations following nsPEF treatment (Figure 4B), which was quantitatively confirmed by a significant elevation in the percentage of non-viable apoptotic cells compared with control (Figure 4C) [145]. Morphological analyses of mitochondria revealed profound structural alterations, with fluorescence microscopy showing fragmentation and reduction in mitochondrial networks under nsPEF exposure (Figure 4D) [145]. Quantitative image analysis revealed a decrease in the mean area, perimeter, form factor, and aspect ratio of mitochondria compared to untreated controls (Figure 4E), collectively confirming mitochondrial shrinkage, rounding, and depolarization [145].

This study demonstrates that nsPEFs act as strong initiators of the mitochondrial apoptosis pathway, linking morphological disruption with programmed cell death [145,146,147]. Although the work was performed in a non-cancerous myocardial model, the findings underscore mitochondria as primary targets of nsPEFs and suggest strong translational potential for oncology. Future studies should extend this approach to malignant cells and evaluate whether combining nsPEFs with pro-apoptotic agents could enhance therapeutic selectivity.

**Figure 5:**
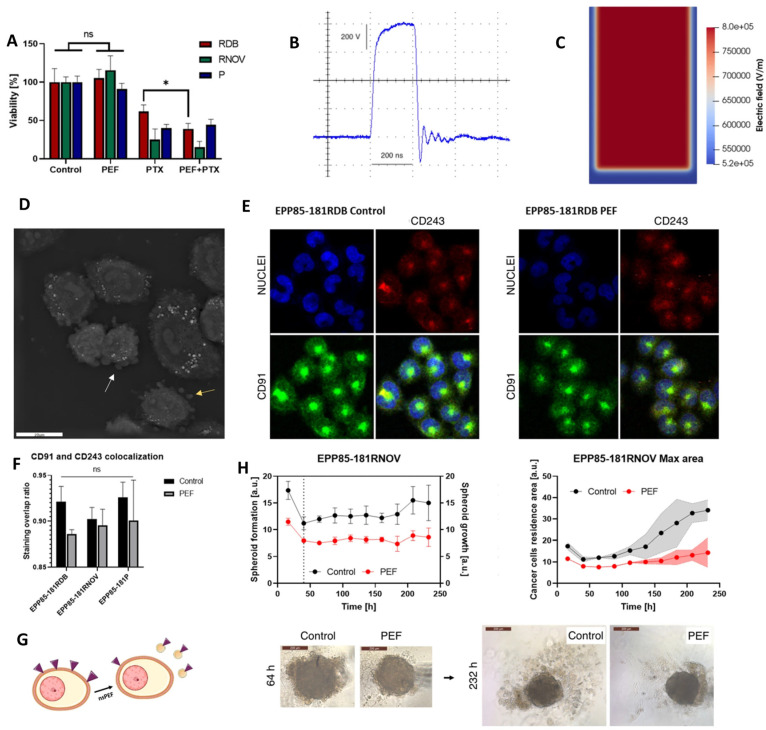
nsPEF modulation of multidrug resistance and tumor spheroid dynamics in pancreatic cancer cells. (**A**) Cell viability following nsPEF, paclitaxel, and combined treatment. (**B**) Representative nsPEF waveform. (**C**) Electric field distribution in culture. (**D**) Cytoskeletal remodeling visualized by microscopy. (**E**,**F**) Altered CD91 and CD243 localization after nsPEF exposure. (**G**) Spheroid cultures showing impaired expansion after treatment. (**H**) Quantification of spheroid growth and cadherin-related adhesion changes. Adapted from [148]. (Differences between groups are denoted by * *p* < 0.05 and ns: non-significant).

Szlasa et al. (2023) investigated how nsPEFs modulate multidrug resistance (MDR) and cytoskeletal dynamics in pancreatic cancer models, providing new insight into their role in sensitizing tumors to chemotherapy [148]. As shown in Figure 5A, cell viability assays demonstrated that paclitaxel (PTX) combined with nsPEF reduced survival in resistant pancreatic cancer cell lines (EPP85-181RDB, EPP85-181RNOV) compared with either treatment alone. The pulse waveform and electric field distribution used for stimulation are depicted in Figure 5B,C [148]. Morphological analyses revealed cytoskeletal remodeling (Figure 5D) and altered localization of CD91 and CD243 transport proteins following nsPEF exposure (Figure 5E,F), indicating changes in the drug efflux machinery. Functional assays further confirmed that nsPEFs impaired spheroid growth and reduced cell adhesion, resulting in a decrease in spheroid size over time and disruption of cadherin organization [148]. Finally, three-dimensional culture models showed that nsPEF treatment significantly limited spheroid expansion after 232 h compared with controls (Figure 5G) [148].

This research is notable because it extends the application of nsPEFs beyond ablation to the modulation of resistance pathways, showing that MDR protein expression is transiently reduced and drug sensitivity enhanced [148,149,150,151]. The findings underscore that nsPEFs are not only physical disruptors of membranes but also modulators of intracellular trafficking and adhesion dynamics [150]. However, the effect on paclitaxel sensitivity was short-lived, lasting less than 48 h, raising questions about the durability of this strategy in clinical settings. Future studies should therefore explore repeated or fractionated nsPEF regimens, combine nsPEFs with sustained-release chemotherapeutics, and determine whether similar MDR suppression occurs in vivo across different tumor types [148,152]. Such approaches could unlock nsPEFs as adjuvants that potentiate chemotherapy in otherwise drug-refractory cancers.

Collectively, cross-study comparisons reveal that nsPEF-induced outcomes depend not only on pulse width and amplitude but also on intrinsic cellular electrophysiology. For example, Fan et al. [145]. demonstrated pronounced mitochondrial depolarization in cardiomyocytes exposed to 100 ns pulses at 10 kV/cm, yet these findings do not translate directly to oncologic models due to the distinct metabolic polarization of tumor mitochondria. In contrast, Szlasa et al. [148]. observed transient MDR suppression (<48 h) in colon carcinoma cells, suggesting that repeated or fractionated pulsing may be required to sustain chemosensitization. Such differences emphasize that nsPEF effects are context-dependent and highlight the need to tune parameters relative to tissue bioelectric profiles rather than applying uniform pulse prescriptions.

Despite broad consensus that nsPEFs permeabilize both plasma and organelle membranes, discrepancies persist regarding the dominant primary target. Mitochondrial depolarization appears central at 100–200 ns, whereas ER and nuclear membrane responses become pronounced at shorter durations (<50 ns). Synthesizing these findings, we propose three mechanistic domains: (i) an organelle-selective regime favoring mitochondria (100–300 ns, 10–20 kV/cm), (ii) an apoptosis-dominant regime balancing plasma and internal poration (50–100 ns), and (iii) an immune-priming regime where ultrashort pulses (<20 ns) trigger ICD through oxidative stress and calcium signaling.

**Figure 6:**
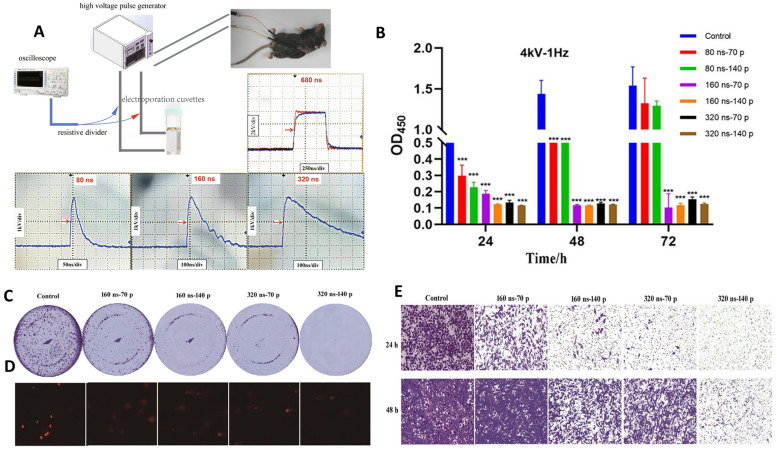
nsPEFs inhibit proliferation, colony formation, and migration in pancreatic adenocarcinoma cells. (**A**) Experimental setup for pulse delivery in vitro and in vivo. (**B**) Cell viability assays showing significant inhibition under 80–320 ns pulses at varying repetition numbers. (**C**) Colony formation assays with dose-dependent suppression of clonogenicity. (**D**) Fluorescent imaging of DNA damage, showing increased nuclear fragmentation in treated cells. (**E**) Migration assays demonstrating reduced cell motility at 24 and 48 h post-exposure. Reused with permission from [153]. (Differences between groups are denoted by *** *p* < 0.001).

Recently, Liang et al. evaluated the antitumor efficacy of nsPEFs in pancreatic adenocarcinoma using both in vitro and in vivo models, revealing strong inhibitory effects on proliferation, colony formation, and metastatic potential [153]. The experimental system (Figure 6A) combined a high-voltage pulse generator with electrode delivery, allowing controlled exposure of Panc02 cells and tumor-bearing mice to nanosecond pulses of varying durations [153]. Cell proliferation assays showed a marked reduction in viability across multiple pulse regimens, with the strongest suppression observed at 160 and 320 ns exposures (Figure 6B) [153]. Long-term clonogenic assays further confirmed the inhibitory effect, as colony numbers decreased significantly with higher pulse intensities (Figure 6C) [153]. Parallel imaging of DNA damage revealed a dose-dependent increase in nuclear fragmentation and fluorescent foci indicative of double-strand breaks (Figure 6D) [153]. Finally, trans-well migration assays demonstrated that nsPEF-treated cells exhibited reduced motility after both 24 and 48 h, underscoring the ability of these pulses to impair metastatic potential (Figure 6E) [153,154].

These findings are important because they demonstrate that nsPEFs not only ablate tumor cell viability but also suppress long-term clonogenic survival and metastatic capacity, two hallmarks of aggressive pancreatic cancer [153,155,156,157]. The study is further strengthened by its use of in vivo mouse models, where nsPEFs achieved tumor growth inhibition without major toxicity to surrounding tissues [153]. However, while the results are promising, the study does not fully resolve how different pulse durations engage distinct molecular pathways. Future work should integrate transcriptomic and proteomic profiling to clarify whether nsPEF-mediated suppression of metastasis is primarily due to cytoskeletal remodeling, gene expression changes, or immune-mediated effects [153]. Moreover, translation into clinical oncology will require systematic optimization of pulse regimens that balance tumor control with preservation of normal pancreatic tissue function.

**Figure 7:**
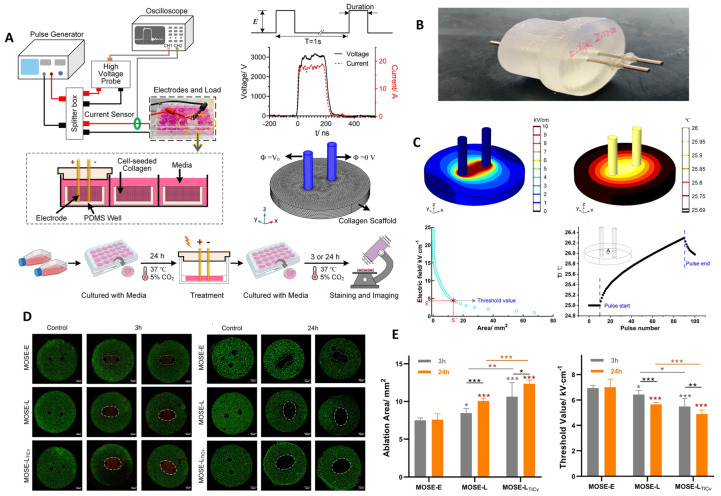
Experimental and mechanistic analysis of nsPEF-induced ablation in ovarian cancer cells. (**A**) Experimental workflow showing pulse generator, electrode configuration, and treatment schedule for cells seeded in 3D collagen scaffolds. (**B**) Custom electrode used for scaffold experiments. (**C**) Simulation of electric field distribution and temperature profiles, confirming electroporation thresholds and non-thermal conditions. (**D**) Fluorescence imaging showing differential ablation across MOSE cell lines with increased sensitivity in malignant and aggressive phenotypes. (**E**) Quantitative analysis of ablation area and lethal thresholds demonstrating stage-dependent susceptibility to nsPEFs. Reused with permission from [158]. (Differences between groups are denoted by * *p* < 0.05, ** *p* < 0.01, and *** *p* < 0.001).

Liu et al. developed a three-dimensional collagen scaffold model to investigate the selective ablation of ovarian cancer cells by nsPEFs [158]. The experimental setup is depicted in Figure 7A, showing the pulse generator and electrode arrangement used to deliver 200 ns pulses to cell-seeded scaffolds, along with a schematic of the treatment and imaging workflow [158]. The custom-built electrode used for these experiments is shown in Figure 7B, which allowed uniform delivery of pulses within the collagen construct. Simulations of the electric field distribution and thermal effects (Figure 7C) revealed that lethal thresholds for electroporation decreased as the number of pulses increased, with negligible thermal rise (<1.5 °C), confirming that observed effects were non-thermal in nature. Fluorescence imaging of cell viability (Figure 7D) demonstrated progressive ablation areas that expanded over 24 h in malignant MOSE-L and highly aggressive MOSE-LTICv cells, whereas benign MOSE-E cells remained largely unaffected [158]. Quantitative analysis (Figure 7E) confirmed that the ablation area increased with malignancy stage, while the lethal threshold required for ablation was significantly lower in aggressive cancer cells compared with benign ones [158].

These findings provide compelling evidence that nsPEFs can exploit the bioelectric vulnerabilities of aggressive tumors, selectively targeting late-stage ovarian cancer cells while sparing non-malignant counterparts [158,159,160]. This stage-specific ablation effect highlights the promise of nsPEFs for preventing recurrence by eliminating cancer stem-like or therapy-resistant populations [158]. However, the study is limited to ex vivo models, and further validation in orthotopic ovarian cancer systems will be crucial to confirm clinical relevance. Future directions include integrating nsPEFs with chemotherapy or microtubule inhibitors (as the authors began testing with Nocodazole) to further enhance tumor selectivity.

**Figure 8:**
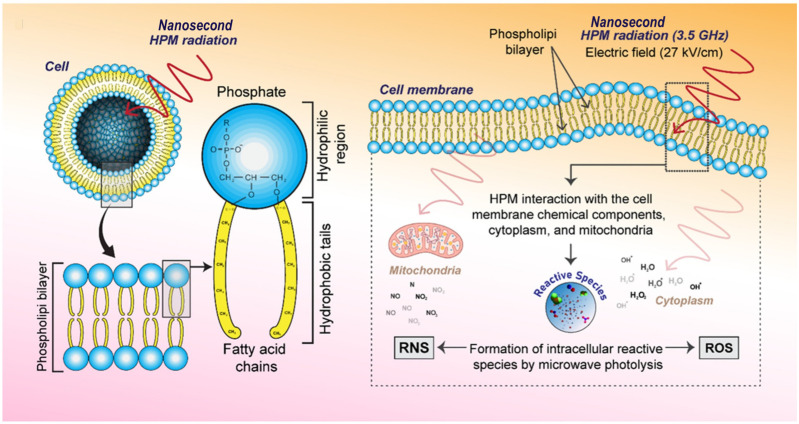
Possibility of the formation of intracellular ROS/RNS by nsPEF associated with pulsed HPM irradiation. When the nsPEF of pulsed HPM interacted with cell membrane components (chemical composition), cytoplasm, and mitochondria, short and long-lived reactive species formed. Adopted from [31].

According to Rana et al. [31], when nsPEF interacts with intracellular systems and the outer membrane, it may produce ROS and RNS species, which play a key role in the cellular effects (Figure 8). Additionally, they discovered that ROS/RNS formed within the liquid, which later entered the cells due to compromised cell permeability caused by electroporation.

**Figure 9:**
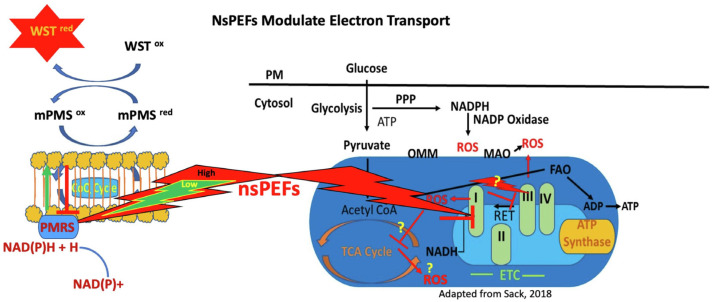
Schematic of nsPEF-induced modulation of plasma membrane redox systems (PMRS) and mitochondrial electron transport chain (ETC), highlighting differential ROS production pathways. Reused with permission from [107].

Moreover, Asadipour et al. reported how nsPEFs modulate electron transport across both the plasma membrane and mitochondria [107]. The study demonstrated that nsPEFs dynamically alter trans-plasma membrane electron transport (tPMET) and mitochondrial electron transport chain activity, resulting in differential ROS generation in cancer versus non-cancer cells (Figure 9). These findings suggest that nsPEFs can selectively exploit redox vulnerabilities, offering a strategy to stress malignant cells while sparing normal tissue [107].

**Figure 10:**
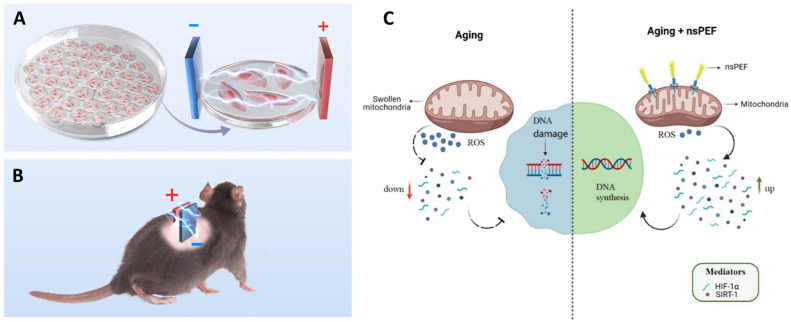
Anti-aging effects of nsPEFs in vitro and in vivo. (**A**) Electrode setup for endothelial cell culture treatment. (**B**) nsPEF stimulation applied to the dorsal skin of aged rodents. (**C**) Mechanistic model showing how nsPEFs restore mitochondrial–nuclear signaling by decreasing ROS, enhancing mitochondrial function, and upregulating HIF-1α/SIRT1 to reverse aging phenotypes. Reused with permission from [7].

Yin et al. demonstrated that nsPEFs can counteract cellular and tissue aging by restoring mitochondrial–nuclear communication [7]. As shown in Figure 10A,B, both in vitro endothelial cultures and aged rodents were treated with daily low-intensity nsPEF exposures. The results revealed a reduction in ROS production, an enhancement of mitochondrial membrane potential, and a decrease in senescence-associated β-gal activity, accompanied by improved proliferation and angiogenic potential. Mechanistically, nsPEFs upregulated HIF-1α and SIRT1, mediators of mitochondrial retrograde signaling, thereby reversing hallmarks of aging (Figure 10C) [7].

These findings are significant because they extend nsPEF applications beyond oncology into the domain of regenerative medicine, suggesting that bioelectric modulation could rejuvenate vascular endothelium and enhance tissue repair [7,161,162,163]. Recent effects of nsPEF were summarized in Table 1. However, further investigation is needed to determine whether such rejuvenating effects are sustainable long-term and whether similar outcomes can be achieved in human vascular aging and other age-related pathologies.

## 4. Innovative Frontiers in nsPEF Therapy

### 4.1. Integration with Nanoparticle-Mediated Drug Delivery

The convergence of nsPEFs with nanotechnology offers a powerful strategy to overcome one of the most persistent challenges in oncology: the efficient, targeted, and intracellular delivery of therapeutic agents [170,171,172]. While nsPEFs alone can permeabilize cellular and organelle membranes, their effects are transient and non-specific. Nanoparticles (NPs), by contrast, can be engineered with surface ligands, stimuli-responsive coatings, and controlled-release properties, but often suffer from limited tumor penetration and intracellular uptake [173]. The integration of nsPEFs with NP-based systems thus represents a synergistic platform where physical membrane poration and molecular targeting cooperate to maximize therapeutic efficacy. Recent studies demonstrate that nsPEFs can significantly enhance the uptake of drug-loaded NPs into tumor cells by increasing transient membrane permeability and disrupting endocytic vesicles. For example, gold and iron oxide NPs have been shown to accumulate more effectively within cancer cells when combined with pulsed electric fields, leading to improved cytotoxicity at lower drug doses [174]. Similarly, polymeric and liposomal nanocarriers encapsulating chemotherapeutics such as doxorubicin or paclitaxel achieve higher intracellular concentrations and more durable retention when administered in conjunction with nsPEF exposure [174]. Importantly, nsPEFs can also facilitate NP penetration beyond the plasma membrane, enabling release of payloads directly into mitochondria or the nucleus—organelles that are otherwise difficult to target pharmacologically [118]. The use of multifunctional NPs further expands this therapeutic landscape. Magnetic or plasmonic NPs allow real-time imaging and tracking of nsPEF-induced delivery, creating theranostic platforms that combine therapy with monitoring. Moreover, stimuli-responsive systems, such as pH-sensitive or ROS-sensitive NPs, can exploit the intracellular microenvironment altered by nsPEFs (e.g., increased Ca^2+^, ROS bursts) to trigger on-demand drug release. This dual responsiveness enhances spatial and temporal precision, aligning with the goals of personalized oncotherapy. From a translational perspective, combining nsPEFs with NP carriers offers two major benefits: dose minimization and resistance circumvention. Lowering the systemic drug burden reduces off-target toxicity, while direct intracellular NP release bypasses membrane pumps and enzymatic degradation that drive chemoresistance. However, critical challenges remain. Optimizing NP size, charge, and composition to synchronize with pulse parameters is still an open question, and in vivo biodistribution studies are limited. Furthermore, the immunological consequences of combining nsPEFs with immune-modulatory NPs, such as checkpoint inhibitor-loaded carriers, have yet to be fully explored [170,175].

**Figure 11:**
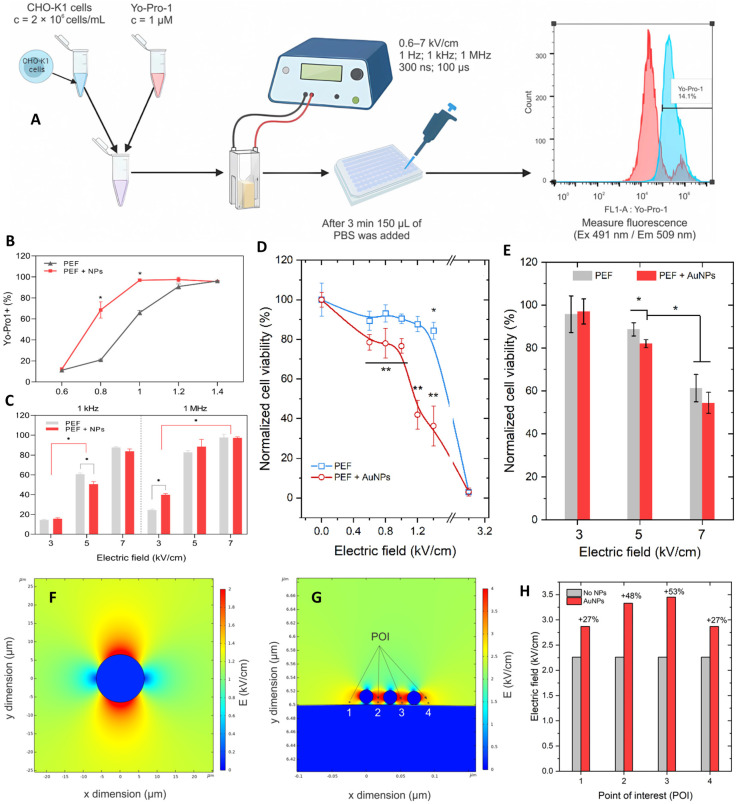
Gold nanoparticle-assisted electropermeabilization under µs and nsPEF conditions. (**A**) Experimental workflow for cell treatment and flow cytometry analysis. (**B**,**C**) Yo-Pro-1 uptake showing enhanced permeabilization with AuNPs. (**D**,**E**) Viability assays demonstrating reduced survival under higher fields with AuNPs, especially for µs pulses. (**F**,**G**) Electric field simulations showing localized intensification by AuNPs. (**H**) Quantification of point-of-interest field enhancement (20–40%) in the presence of AuNPs. Adapted from [174]. (Differences between groups are denoted by * *p* < 0.05 and ** *p* < 0.01).

Radzevičiūtė-Valčiukė et al. [174] explored the effects of gold nanoparticles (AuNPs) on electropermeabilization and viability under both microsecond and nanosecond pulse regimens. As shown in Figure 11A, CHO-K1 cells were treated with AuNPs and subjected to either µs or nsPEF exposures, followed by Yo-Pro-1 uptake analysis [174]. Figure 11B demonstrates that PEF + AuNPs enhanced membrane permeabilization compared with PEF alone, while Figure 11C confirms this effect across different field strengths and frequencies. Cell viability assays (Figure 11D,E) revealed that AuNPs reduced survival under higher field strengths, particularly when combined with µs protocols, while nsPEFs maintained relatively higher viability [174]. Field distribution simulations (Figure 11F,G) further illustrated how AuNPs locally intensified electric fields, a finding quantified in Figure 11H, where point-of-interest measurements confirmed increased field strength by 20–40% with AuNP presence [174].

These results highlight that AuNPs can amplify local electric fields and enhance electropermeabilization efficiency, but this comes at the cost of reduced viability under µs pulse conditions. Interestingly, nsPEFs showed a more favorable balance, maintaining lower ROS induction and better viability despite AuNPs [174]. From an expert perspective, this suggests that future optimization of nanoparticle surface chemistry and nsPEF parameters could unlock safe and effective synergy, particularly for applications in gene delivery or targeted oncotherapy where localized permeabilization without excessive cytotoxicity is critical.

**Figure 12:**
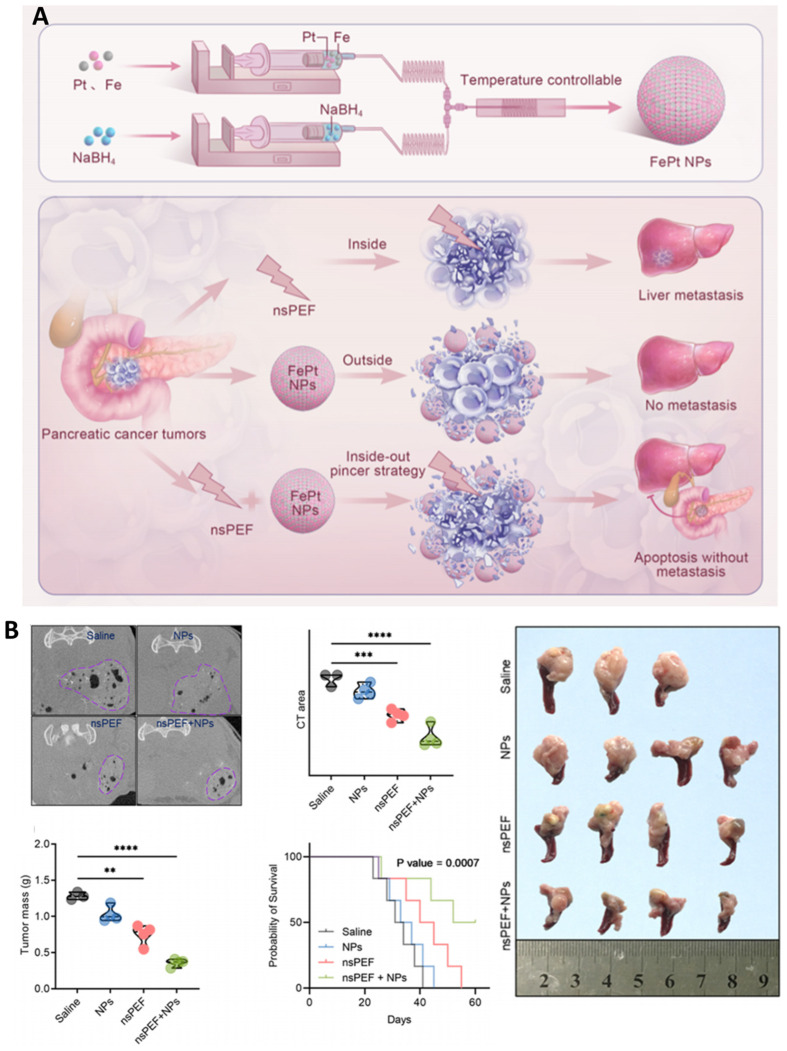
FePt nanoparticle-enhanced nsPEF therapy in pancreatic cancer. (**A**) Schematic of FePt NP synthesis and “inside-out” strategy showing how nsPEFs promote NP penetration, preventing recurrence and metastasis. (**B**) In vivo validation: CT imaging contrast improved, tumor burden decreased, survival extended, and metastasis suppressed in the nsPEF + FePt NP group. Reused with permission from [118]. (Differences between groups are denoted by ** *p* < 0.01, *** *p* < 0.001, and **** *p* < 0.0001).

Hu et al. (2025) developed a theranostic strategy combining nsPEFs with FePt nanoparticles (FePt NPs) for pancreatic cancer treatment [118]. As illustrated in Figure 12A, FePt NPs were synthesized and used in an “inside-out” ablation approach, where nsPEFs facilitated NP uptake into both the tumor core and periphery, thereby reducing the risk of recurrence and metastasis. In vivo results (Figure 12B) confirmed that FePt NPs enhanced CT imaging contrast, significantly reduced tumor mass, and extended the survival of treated mice compared with saline, NP-only, or nsPEF-only groups [118]. Importantly, the combination strategy inhibited liver metastasis, while histological analysis revealed apoptosis without significant off-target toxicity. These findings highlight the dual benefit of FePt NPs as both catalytic enhancers of nsPEF-mediated tumor killing and diagnostic agents for CT-based monitoring. From an expert perspective, this work underscores the potential of integrating nsPEFs with multifunctional nanoparticles to achieve not only precise tumor eradication but also real-time imaging, opening pathways for clinical translation in pancreatic cancer.

In summary, the integration of nsPEFs with NP-mediated drug delivery embodies a promising frontier in precision oncology, bridging bioelectric modulation with nanomedicine engineering [170]. The summary of results using combination treatments with nsPEF is summarized in Table 2. By coupling physical electroporation with nanoscale carriers, this approach can achieve deeper tumor penetration, organelle-specific targeting, and combinatorial immune activation. Future work should focus on in vivo optimization, AI-driven modeling of NP–nsPEF interactions, and clinical trials assessing safety and efficacy in drug-resistant cancers.

Toxicity and aggregation—practical constraints. While AuNPs and SPIONs can amplify local E-fields and improve cargo delivery, their in vivo behavior introduces non-trivial risks. Gold colloids commonly exhibit reticuloendothelial system (RES) uptake with hepatic and splenic accumulation and long tissue residence times, and highly cationic surfaces can trigger complement activation, hemolysis, and coagulation perturbations. SPIONs share RES sequestration and pose iron-overload and oxidative stress risks through Fenton chemistry, particularly with high cumulative dosing: they may also generate MRI artifacts that complicate response assessment. Critically, NP aggregation in the TME (driven by ionic strength, proteins, and ECM binding) reduces penetration, distorts local E-field enhancement, and can yield non-uniform nsPEF effects. To mitigate these issues, we favor near-neutral potential (≈−10 to +5 mV), PEG or zwitterionic coatings (e.g., DSPE-PEG, phosphorylcholine), low polydispersity (PDI < 0.2), membrane cloaking to limit opsonization, and size windows of ~20–80 nm to balance penetration with clearance. Aggregation control should be validated in serum and ECM-mimetic media (DLS/NTA), with in vivo quantification by MRI (SPIONs) or CT/ICP-MS (AuNPs) to link intratumoral dose to effect. Given these constraints, intratumoral or loco-regional administration is preferable when feasible, reserving systemic delivery for constructs with validated stability, clearance, and immunocompatibility. Finally, because clustered NPs can create E-field “hot spots”, nsPEF protocols should be tuned to exploit on-target amplification while avoiding off-target deposition, an argument for image-guided dosing and feedback-controlled pulse delivery in NP-assisted regimens.

### 4.2. Integration of nsPEFs with Drugs

**Figure 13:**
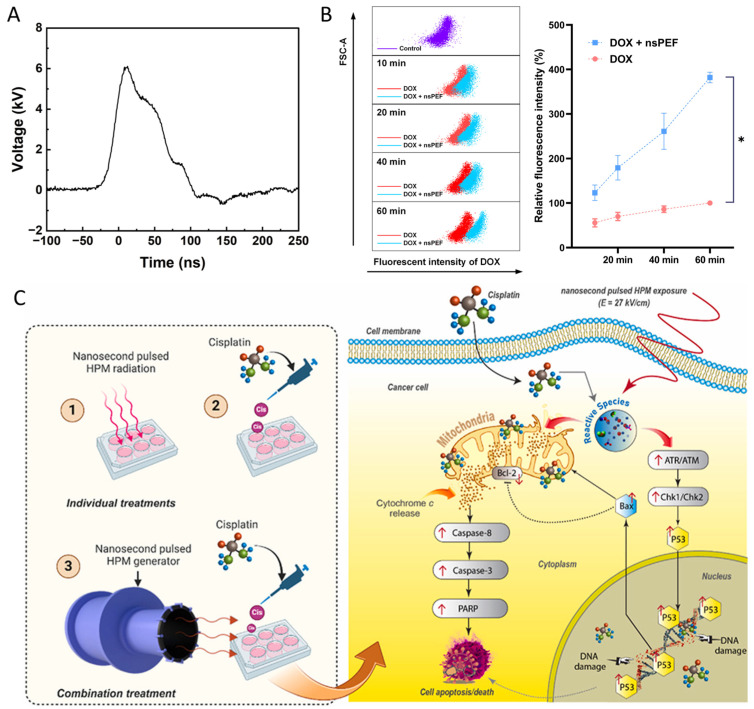
(**A**) The waveform of the pulse delivered to cuvettes. (**B**) nsPEF consistently enhanced intracellular DOX uptake after treatment. Adopted from [177]. (**C**) nsPEF from pulsed HPM increases the membrane permeability, and cisplatin uptake increases to induce apoptosis. Reused with permission from [178]. (Differences between groups are denoted by * *p* < 0.05).

While nsPEFs alone are capable of inducing apoptosis, necrosis, and immunogenic cell death, their most promising application lies in synergy with pharmacological agents [176]. Drugs often face barriers such as limited tumor penetration, rapid clearance, and multidrug resistance, whereas nsPEFs transiently permeabilize cellular and organelle membranes, disrupt efflux mechanisms, and alter intracellular signaling pathways. This creates a unique therapeutic window where nsPEFs enhance intracellular drug accumulation, alter drug trafficking, and sensitize resistant cells, thereby improving overall efficacy at reduced systemic doses. Recent studies have provided compelling evidence for such synergy. Ma et al. demonstrated that doxorubicin (DOX) uptake in hepatocellular carcinoma cells was significantly increased when administered after nsPEF exposure, with the strongest effect observed when DOX delivery was delayed by 40 min (Figure 13A,B) [177]. Similarly, Rana et al. reported enhanced cisplatin uptake following nsPEF (27 kV/cm) irradiation of pulsed HPM, leading to higher cell death rates and increased DNA double-strand breaks (Figure 13C) [178].

This regimen not only enhanced apoptosis and mitochondrial damage but also achieved a synergism quotient greater than one, suggesting a true supra-additive effect. Similarly, nsPEFs induce Ca^2+^-dependent mitochondrial apoptosis in lung cancer cells, and when combined with conventional chemotherapeutics, this effect can overcome resistance by amplifying mitochondrial disruption. These findings highlight the importance of temporal sequencing; whether drugs are given before, during, or after nsPEF treatment critically determines therapeutic outcomes.

The superior efficacy of delayed drug administration following nsPEF exposure can be attributed to transient biophysical and biochemical changes that persist after pulsing. Immediately post-nsPEF, the plasma membrane undergoes partial resealing, maintaining selective yet enhanced permeability for small molecules. Concurrently, cytoskeletal softening and increased endocytic flux promote intracellular trafficking, while transient disruption of ATP-dependent efflux pumps (e.g., P-gp) prolongs drug retention. Collectively, these processes create a temporal ‘window of vulnerability,’ typically spanning 30–60 min post-treatment, during which chemotherapeutic agents such as doxorubicin penetrate and accumulate more effectively within tumor cells.

Integration is not limited to classical chemotherapeutics. Targeted therapies and small molecules have also shown potential when paired with nsPEFs. By destabilizing plasma and organelle membranes, nsPEFs lower the threshold for cytotoxicity of tyrosine kinase inhibitors or microtubule-targeting agents, making previously resistant tumor cells more vulnerable. In addition, nsPEFs potentiate the delivery of hydrophilic drugs that typically suffer from poor membrane permeability, allowing for lower doses and reduced off-target toxicity. From a translational perspective, nsPEF–drug combinations offer three key advantages. First, they enable dose minimization, reducing systemic toxicity of potent chemotherapeutics such as DOX [177]. Second, they circumvent resistance mechanisms by bypassing efflux pumps and altering drug metabolism pathways. Third, they expand the therapeutic spectrum, allowing non-permeant or organelle-targeted drugs to reach intracellular compartments that were previously inaccessible [177]. However, major challenges remain, particularly regarding optimization of pulse parameters for each drug class, the risk of cumulative off-target toxicity, and the lack of clinical trials directly testing these strategies. Recent studies indicate that fractionated nsPEF regimens, where pulse trains are temporally spaced to allow drug clearance from normal tissues, can substantially minimize systemic toxicity without compromising antitumor efficacy [148]. Additionally, combining nsPEFs with localized or intratumoral drug delivery platforms, such as liposomal or hydrogel-based systems, further confines drug activity to the targeted region, thereby reducing off-target accumulation and enhancing the therapeutic index.

Actionable design rules for combinations. Three operational insights recur across drug integrations: (i) timing, drug-after windows on the order of 20–60 min can maximize intracellular accumulation and cytotoxic synergy; (ii) microdomain disruption, nsPEF transiently perturbs plasma-membrane microdomains, facilitating uptake of cisplatin-class agents; (iii) field amplification, conductive or catalytic nanoparticles raise local E-fields and enable equivalent biological effect at lower bulk voltages, improving selectivity. Divergences among reports track to NP size/charge and burst repetition (kHz vs. MHz); we therefore propose reporting NP physicochemical descriptors and full waveform metadata as minimal standards for comparability.

Overall, the integration of nsPEFs with drugs exemplifies a rational approach to precision intracellular oncotherapy, where physical membrane poration and pharmacological modulation converge. Future studies should focus on optimizing drug–pulse timing, expanding combinations to targeted and immune-modulatory agents, and conducting systematic in vivo studies to establish safety and efficacy profiles that will pave the way for clinical translation.

**Table 2:** Integration of nsPEFs with drugs and nanoparticle-based systems in cancer models for combination treatments (↑ increase, ↓ decrease).

Tumor/Cell Model	Combination	nsPEF Parameters	Mechanistic Outcomes	Therapeutic Implications	Refs.
Hep3B hepatocellular carcinoma cells	Doxorubicin (0.5 µM, free drug)	100 ns, 15 kV/cm, 400 pulses, 10 Hz	DOX uptake ↑ (delayed 40 min > immediate), Cell viability ↓, Cell cycle arrest ↑, Early apoptosis ↑, Mitochondrial swelling ↑	Timing-dependent synergy: delayed DOX (40 min post nsPEF) most effective (SQ = 1.40 @48 h), enabling low-dose chemo with enhanced cytotoxicity	[177]
Murine pancreatic cancer (Panc02, C57BL/6 mice)	Neutrophil membrane-coated liposomal gemcitabine (NE/Lip-GEM)	300 ns, 24 kV/cm, 100 pulses	NE/Lip-GEM uptake ↑ in nsPEF-treated tumors (3–3.6×), Tumor volume ↓, Ki-67 ↓, Apoptosis ↑, Cytokines (TNF-α, IL-1β, CXCL1) ↑	nsPEFs amplify inflammatory signals → recruit NE/Lip-GEM to tumor; combination markedly enhances pancreatic tumor suppression without systemic toxicity	[179]
Human hepatocellular carcinoma (SMMC7721, BEL7402, HCCLM3)	Cisplatin (5 µg/mL)	100 ns, 40 kV/cm, 12 pulses (1 pulse/min)	Membrane microdomains disrupted ↑, PI uptake ↑, Cisplatin cytotoxicity ↑, Synergy strongest within 2 h, then ↓	nsPEFs enhance cisplatin uptake via microdomain disruption; timing critical for synergy	[180]
Mouse myeloma (Sp2/0) tumors in BALB/c mice	Doxorubicin (12 mg/kg, i.p.)	3.5 kV/cm, 800 ns, 250 pulses (nano-ECT) vs. 1.4 kV/cm, 100 µs, 8 pulses	Tumor growth delay ↑, Necrosis localized ↑, PI uptake ↑, Energy delivered ↓ (15% less vs. ESOPE), Thermal effects negligible	nsPEF-based nano-ECT achieved efficacy comparable to microsecond ESOPE protocols, with better energy control and precise tumor localization	[181]
Human breast (MCF-7/WT, MCF-7/DX) and colon (LoVo, LoVoDX) cancer cells (DOX-sensitive & resistant)	Doxorubicin (2–50 µM)	PEF1: 10 kV/cm × 300 ns × 200; PEF2: 40 kV/cm × 20 ns × 400; PEF3: 60 kV/cm × 20 ns × 400; PEF4: 1.2 kV/cm × 100 µs × 8 (ESOPE)	Viability ↓ (greater at 72 h), IC_50_ ↓ markedly with PEF2/PEF3, ROS ↑, GSH ↓ in resistant cells, Confluency ↓, Mitochondrial alterations ↑	nsPEFs potentiate DOX cytotoxicity, especially in resistant breast/colon cancer cells; ultrashort pulses (20 ns, high field) outperform ESOPE	[182]
CHO-K1 cells (DNA delivery model)	Gold nanoparticles (AuNPs; 9–22 nm, citrate-capped) + pEGFP-N1 plasmid	ESOPE: 100 µs × 8, 0.6–1.4 kV/cm; nsPEF: 300 ns × 100, 3–7 kV/cm, 1 kHz/1 MHz	Electrotransfection ↑ with AuNPs under µs pulses, GFP expression ↑ (up to 30%), ROS ↑ with µs pulses (not nsPEF), Viability ↓ at >1 kV/cm + AuNPs	AuNPs amplify µs-ECT efficiency; nsPEFs remain effective without NPs, offering lower ROS and higher viability	[174]
Human pancreatic cancer (L3.6pl cells; orthotopic mouse model)	FePt nanoparticles (3–5 nm, catalytic, ROS-generating)	300 ns, 25 kV/cm, 30 pulses (2 Hz)	Selective cytotoxicity ↑ in cancer vs. normal cells, ROS ↑ (•OH, O_2_•^−^), O_2_ release ↑, Apoptosis ↑, Proliferation ↓, Tumor mass ↓, CT contrast ↑, Survival ↑ (>70 days vs. 50–65 days controls)	nsPEF + FePt NPs achieve dual-strike therapy: core tumor ablation + NP uptake in periphery, preventing recurrence/metastasis; also provide CT imaging capability for theranostics	[118]
Murine mammary cancer (4T1)	Gold NPs (13 nm) + Bleomycin	µs: 100 µs × 8, 0.6–1.5 kV/cm; ns: 300–700 ns × 100, 6 kV/cm, 1 kHz–1 MHz	Permeabilization ↑, Cytotoxicity ↑, Resealing ↓, Strong synergy at 0.9 kV/cm (µs) and 1 MHz (ns)	AuNPs amplify fields, enabling effective BLM-ECT at lower voltages	[183]

## 5. Challenges and Future Horizons

Although nsPEFs have demonstrated remarkable intracellular precision in controlled experimental settings, their clinical translation remains constrained by three foundational barriers: scalability, standardization, and specificity. These are not peripheral challenges but rather fundamental scientific questions that define the future of the field: How can pulse delivery be scaled safely across complex, heterogeneous tissues? What universal dosimetric framework can translate laboratory protocols into reproducible clinical outcomes? And how can tumor-selective specificity be preserved when electrical fields inherently affect all excitable and non-excitable cells within range?

The nsPEF has crossed the threshold from biophysical curiosity to a programmable modality for intracellular oncotherapy, yet a credible path to front-line use hinges on solving four intertwined problems: specificity, scalability, standardization, and synergy [2,5,184]. Below, we outline where the field stands, what still constrains it, and how the next wave of studies can convert mechanistic promise into durable clinical impact.

### 5.1. Enhancing Specificity While Minimizing Off-Target Effects

At the cellular scale, nsPEF’s greatest strength—multi-organelle access—can also be its liability. Preferential nuclear and ER poration, mitochondrial depolarization, and Ca^2+^ flux are now well documented in malignant models [80,124,153], but these same pathways exist in normal tissue. The central specificity problem is therefore not whether nsPEF can kill cancer cells, but whether it can consistently spare the neighborhood. Three levers are emerging:

Field shaping and microdosimetry. Finite-element models accurately predict E-field hot spots at membranes and organelles [185], but in vivo heterogeneity, fibrosis, edema, and vasculature distort those maps. Translation demands pre-planning and verification workflows: pre-treatment modeling on patient imaging; intra-procedural impedance or ultrasound elastography to update tissue conductivity; and post-pulse readouts (electrical impedance spectroscopy, fast thermometry) to confirm dose. MHz-repetition nsPEF regimens that maintain non-thermal conditions while deepening permeabilization should be prioritized where thermal boundaries are tight (pancreas, bile duct) [166,186].

Bioelectric phenotyping and patient selection. Late-stage and drug-resistant phenotypes exhibit lower lethal thresholds and larger ablation zones under identical parameters [187]. Incorporating ex vivo electrical phenotyping (mini-biopsy slabs and rapid viability maps under test pulses) could stratify patients to parameter sets before therapy. In parallel, response biomarkers, ΔΨm collapse kinetics, ICD surrogates, PD-1/MHC-II shifts [188,189], and sphingolipid-programmed myeloid differentiation, can inform real-time “stay-or-escalate” decisions.

Adjuncts that narrow the target. Two directions are maturing: calcium-assisted protocols that exploit malignant Ca^2+^ handling [190] and conductive/amplifying nanoparticles that locally raise the E-field to therapeutic thresholds at lower bulk voltages. The engineering mandate is to keep the on-target:off-target field ratio high by combining conformal electrodes with micro- or nano-scale field boosters inside the tumor.

### 5.2. Scalability for Clinical Adoption

Scalability represents another major hurdle. Bench-top protocols often rely on idealized conditions, whereas clinical scenarios involve organ motion, blood perfusion, variable tissue conductivity, and the need for rapid, image-guided procedures [191]. The first pilot clinical studies in hepatocellular carcinoma demonstrate safety and encouraging local control, but widespread adoption will require industrial-grade pulse generators capable of delivering high-frequency, low-jitter waveforms and electrode systems that can conform to irregular tumor geometries while remaining stable during physiological movement [192]. Equally important is the development of standardized protocol libraries tailored to different tumor types and anatomical sites. Unlike thermal ablation techniques, nsPEF protocols vary significantly in pulse width, amplitude, repetition rate, and total energy delivered, resulting in inconsistent outcomes across laboratories. Establishing universally accepted dosimetry and quality assurance standards, analogous to the ESOPE guidelines for microsecond electroporation, will be crucial for ensuring reproducibility and facilitating regulatory approval.

### 5.3. Synergy: Immunotherapy, Drugs, and Nanotechnology

Another central challenge lies in optimizing combination regimens [193]. nsPEFs enhance intracellular uptake of chemotherapeutics, destabilize membrane microdomains to facilitate cisplatin entry, and potentiate doxorubicin accumulation when administered in carefully timed sequences [178,194,195]. The timing of drug delivery relative to pulsing has emerged as a decisive factor, with delayed administration in some cases producing the greatest synergy. Similarly, integration with calcium has demonstrated remarkable efficacy in overcoming resistance in otherwise refractory gastric and colorectal cancer cells, while nanoparticle-assisted nsPEFs have amplified local fields, catalyzed ROS, and introduced theranostic capabilities. Yet these synergies also introduce new questions regarding optimal sequencing, dosing, and safety [196]. Few studies have systematically compared different pulse–drug schedules or evaluated pharmacokinetics in vivo, and no clinical trial has yet prospectively tested nsPEFs in combination with immune checkpoint inhibitors, despite preclinical data showing enhanced PD-1 and MHC-II expression and durable T-cell memory.

Although nsPEFs are often described as programmable tools for precision intracellular modulation, this programmability remains an emerging property rather than an established control paradigm [124]. Translating electrical waveform design into predictable biological outcomes is complicated by inter-tissue variability in conductivity, field distribution, and repair kinetics. Similarly, while nsPEF-induced immunogenic cell death promotes immune activation, excessive or poorly tuned exposures may trigger counterproductive inflammation or transient immunosuppression. Thus, the immunomodulatory potential of nsPEFs represents a double-edged sword—capable of priming anti-tumor immunity or, under certain conditions, dampening it through T-cell exhaustion and cytokine dysregulation. Achieving true programmability will depend on closed-loop feedback systems that couple real-time electrical and biological readouts to maintain nsPEF exposures within immunologically favorable thresholds [124,197].

In vitro demonstrations of nsPEF ‘programmability’ rely on homogeneous electric field environments that do not fully capture the biophysical complexity of clinical tissues. In vivo, heterogeneity in conductivity caused by perfusion gradients, fibrosis, and edema can alter local field intensities by more than an order of magnitude, leading to spatially uneven permeabilization [124]. Thus, rather than a solved characteristic, programmability should be viewed as an engineering frontier requiring integration of real-time dosimetry, adaptive feedback, and closed-loop control systems capable of dynamically adjusting pulse delivery to local tissue conditions.

The immunological consequences of nsPEF are equally complex. Although nsPEF-induced immunogenic cell death exposes tumor antigens and promotes dendritic activation, several studies also report concurrent upregulation of checkpoint receptors such as PD-1 and PD-L1 on T cells and tumor cells [116,197]. This duality suggests that nsPEF can simultaneously ‘ignite’ and ‘regulate’ the immune response. Therefore, combining nsPEFs with immune checkpoint inhibitors is not merely synergistic, it may be essential to sustain the anti-tumor immune cascade and prevent feedback-driven immune exhaustion. Understanding and controlling this balance will be pivotal to translating nsPEF immunomodulation from transient activation to durable systemic immunity [197].

### 5.4. Beyond Oncology: System Effects and Rejuvenation

nsPEF’s ability to modulate redox systems and mitochondrial-nuclear signaling has implications beyond tumors. Endothelial rejuvenation via HIF-1α/SIRT1 and vascular functional recovery has been shown in vitro and in aged rodents [7,140]. While outside the main oncologic scope, these data reinforce that nsPEF is a system modulator: oncology protocols must therefore surveil tissue-level benefits and risks (wound healing, fibrosis, vascular tone) in parallel with tumor endpoints.

### 5.5. A Pragmatic Roadmap

The following research priorities are not merely developmental milestones but direct experimental responses to the core translational barriers identified above. For instance, the deployment of closed-loop generators with impedance and temperature feedback is intended to resolve the programmability paradox by coupling real-time tissue readouts to adaptive waveform control. Similarly, standardized waveform metadata and inter-laboratory reference phantoms aim to overcome the lack of cross-platform reproducibility, while biomarker-anchored clinical trials, tracking ΔΨm kinetics, ICD markers, and immune memory formation, are designed to define and validate tumor-selective specificity in human tissues.

Near-term (12–24 months): finalize indication-specific parameter libraries, adopt reporting standards, and run pilot combination trials (nsPEF-first + PD-1, nsPEF + timed DOX, nsPEF + Ca^2+^) with immune and pharmacologic endpoints. Mid-term (2–4 years): deploy closed-loop generators with impedance/temperature feedback; integrate AI-guided planning using shared datasets; and scale conformal electrodes for pancreas/liver with endoscopic or percutaneous access. Long-term (4–6 years): validate theranostic NP-assisted nsPEF in one solid tumor; and launch multicenter RCTs versus thermal ablation, using recurrence-free survival and quality-of-life co-primaries.

## 6. Conclusions

The nsPEF is redefining how energy can be used as medicine. nsPEF is no longer a laboratory curiosity: it is a programmable, intracellular therapy. Its value stems from time-resolved access to the inside of the cell, where mitochondria depolarize, ER Ca^2+^ stores discharge, and the nuclear envelope becomes transiently permeable. These concurrent perturbations form a redundant kill network that malignant cells struggle to evade, explaining consistent apoptosis and ICD across models, including drug-resistant disease. A coherent design grammar is now visible. Sub-10 ns pulses bias effects to internal membranes; conversely, 100–300 ns pulses couple robust plasma-membrane poration with organelle stress. Amplitude and pulse number set the apoptosis–necrosis boundary, while rise time and spectral content determine how effectively the plasma-membrane capacitor is bypassed. These rules enable rational recipes: collapse tumor bioenergetics, prime in situ vaccination, or ablate margins without thermal injury.

The most compelling path forward is combination treatments. nsPEFs boost intracellular drug uptake, restore sensitivity in resistant cells, and generate precise timing windows for synergy with drugs. Calcium co-delivery and nanoparticle amplification further enhance efficacy, while recent studies show nsPEFs modulate checkpoint receptors and reprogram myeloid cells, positioning them as partners for immunotherapy rather than stand-alone ablation.

Finally, the horizon extends beyond oncology. The same ability to tune redox and mitochondrial–nuclear signaling that powers tumor control also hints at applications in vascular rejuvenation and tissue repair. That breadth is an opportunity and a caution, as rigorous long-term safety, biodistribution (for nanoparticle-assisted regimens), and genotoxicity surveillance are essential to preserve a wide therapeutic window. With rigorous engineering and biomarker-anchored trials, nsPEFs could evolve into a first-line pillar of cancer therapy, redefining how electricity is used as medicine.

## Figures and Tables

**Table 1:** Summary of recent studies investigating nsPEFs in cancer and related biological models. The table highlights tumor/cell models, pulse parameters, primary mechanistic outcomes (↑ increase, ↓ decrease), and therapeutic implications, illustrating how nsPEFs induce apoptosis, necroptosis, immunomodulation, redox modulation, and clinical translation potential across different systems.

Tumor Model	Pulse Parameters	Primary Outcomes	Therapeutic Implications	Ref.
Human breast cancer cells (MDA-MB-231)	5 ns, 70–100 kV/cm, 2000–10,000 pulses, 100 Hz	Nuclear pores ↑, ER Ca^2+^ release ↑, Mitochondrial depolarization ↑, Cytoskeleton integrity ↓	Apoptosis ↑, ICD ↑, Therapy resistance ↓	[80]
Rat myocardial cells (H9C2)	100 ns, 20 kV/cm, 80 pulses	Mitochondrial membrane potential ↓, Mitochondrial area ↓, Perimeter ↓, Apoptosis ↑	Initiates mitochondrial apoptosis pathway ↑, Non-thermal ablation potential ↑, Tissue selectivity ↑	[145]
Human pancreatic cancer cells (EPP85-181RDB, EPP85-181RNOV, EPP85-181P)	200 ns, 8 kV/cm, 100 pulses, 10 kHz	MDR protein (P-gp, LRP) expression ↓, Microvesicle release ↑, Actin remodeling ↑/↓ (cell line-dependent), Spheroid growth ↓, Cadherin expression ↓	MDR ↓, Paclitaxel sensitivity ↑ (transient), Tumor growth ↓, Cell adhesion ↑	[148]
Murine pancreatic adenocarcinoma (Panc02 cells, C57BL/6J mice)	80–320 ns (cells), 160 ns/4 kV/70–280 pulses/1 Hz (in vitro); 680 ns/6 kV/400 pulses/1 Hz (in vivo)	Cell viability ↓, Colony formation ↓, Migration ↓, DNA damage ↑, Tumor growth ↓, Gene expression changes ↑ (CDK1, CENPA, UBE2C, etc.), ceRNA network altered	Tumor suppression ↑, Metastasis ↓, No organ toxicity, Prognostic genes identified for pancreatic cancer	[153]
Breast cancer cells (MCF-7, MDA-MB-231)	50 ns, 20 kV/cm, 2 kHz, 120 s	MCF-7: Apoptosis ↑ (Annexin V ↑, caspase-3/7 ↑, ΔΨm ↓, ROS ↑, NADH lifetime ↑). MDA-MB-231: Necroptosis ↑ (plasma membrane rupture ↑, nuclear swelling ↑, caspase-independent, NADH lifetime ↓, Ca^2+^ dysregulation ↑)	Subtype-specific responses: apoptosis in hormone-positive cells ↑, necroptosis in triple-negative cells ↑, therapy resistance ↓	[164]
Mouse ovarian surface epithelial cells (MOSE-E: benign, MOSE-L: malignant, MOSE-LTICv: highly aggressive)	200 ns, 15 kV/cm, 80 pulses, 1 Hz, 3D collagen scaffolds ± Nocodazole	Ablation area ↑ with malignancy stage (MOSE-LTICv > MOSE-L > MOSE-E), Lethal threshold ↓ with malignancy stage, Ablation area stable in benign cells but ↑ in malignant/aggressive cells over 24 h, Thermal rise negligible (<1.5 °C)	Selective ablation of late-stage/aggressive cells ↑, Tumor recurrence ↓, Benign tissue sparing ↑, Combination with Nocodazole further ↑ ablation	[158]
H9c2 cardiac myoblasts (non-cancer) and 4T1-luc breast cancer cells	60 ns, 40 kV/cm, 1 Hz, 1–75 pulses (charging effect up to 0.02 Vs/cm)	Biphasic modulation of plasma tPMET ↑ (low) ↓ (high), ETS I ↓, H9c2: mROS ↑ cROS ↓, 4T1: cROS ↑ mROS ↑	Cancer adaptation via glycolysis ↑, Normal cell stress ↑, Redox selectivity exploitable	[107]
Human hepatocellular carcinoma (HepG2) cells	300 ns, 10–30 kV/cm, 1 Hz, 200 pulses	Cell viability ↓ (dose-dependent), ROS ↑, ΔΨm ↓, Apoptosis ↑ (Annexin V, caspase-3/7), Necrosis ↑ at higher fields	nsPEFs trigger apoptosis at moderate fields and necrosis at high fields; potential for dose-controlled ablation in liver cancer	[165]
Human melanoma (C32, A375)	200 ns, 100 pulses, 4–16 kV/cm, 10 kHz	Viability ↓ (C32: to 51%, A375: to 66% at 16 kV/cm), Membrane permeabilization ↑, Morphological changes (contraction, vesiculation, lipid redistribution ↑), PD-1 expression ↑, MHC-II expression ↑, Cytokine secretion: TNF-α ↑, IL-6 ↑/↓ (cell line dependent), IL-1β ↑	nsPEFs modulate immune checkpoint receptors and cytokines, enhancing antitumor immunity and supporting synergy with checkpoint inhibitors (PD-1/PD-L1 axis).	[124]
Human lung carcinoma (A549, 2D & 3D spheroids)	200 ns, 100 pulses, 4–16 kV/cm, 10 kHz	Viability ↓, Membrane permeability ↑, PD-1 ↑, MHC-II ↑, Cytokines (TNF-α ↑, IL-1β ↑, IL-6 variable)	nsPEFs enhance immunogenicity and checkpoint pathways → synergy with PD-1/PD-L1 blockade	[166]
CT-26 murine colorectal carcinoma (cells & peritoneal metastasis mice)	10 ns, 1–1000 pulses, 10–200 Hz	Cell death ↑, Membrane permeability ↑, Ca^2+^ influx ↑, Mitochondrial swelling ↑, Tumor regression ↑, CD8^+^ T-cell infiltration ↑	nsPEFs induce cytotoxicity + immune activation → potential for CRC peritoneal metastasis therapy	[12]
Human umbilical vein endothelial cells (HUVECs, D-galactose-induced senescence) & aged rodents (skin)	3 kV/cm, 3 min/day × 14 days (in vitro & in vivo)	SA-β-Gal ↓, ROS ↓, Proliferation (EdU+) ↑, ΔΨm ↑, HIF-1α ↑, SIRT1 ↑, Angiogenesis ↑ in aged skin	nsPEFs rejuvenate aging ECs by restoring mitochondrial–nuclear retrograde communication and promoting vascular regeneration	[7]
Liver cancer patients (clinical, n = 15) & mouse HCC model	300 ns, 30 kV, 800–3200 pulses, 2–3 electrodes	Local RFS (12 mo) ↑ (86.7%), Overall RFS ↑ (60%), ALT/AST transient ↑, Sphingolipid metabolism ↑ in Ly6c2^+^ MNPs, CD8^+^ T-cell activation ↑	nsPEFs safe & effective; efficacy linked to sphingolipid-driven MNP differentiation → durable antitumor immunity	[167]
Human lung cancer (A549)	50 or 100 ns, 20 kV/cm, 2 kHz, microelectrode chip	Apoptosis ↑ (blebbing, PS externalization, caspase-3/7 ↑), Intracellular Ca^2+^ ↑ (ER release, CRAC entry), ROS (O_2_^−^) ↑, NADH lifetime ↑, ΔΨm ↓	nsPEFs induce Ca^2+^-dependent mitochondrial apoptosis, linking ER release, ROS generation, and ΔΨm collapse in lung cancer	[99]
Bovine adrenal chromaffin cells (neuroendocrine model)	Single pulses, 3–50 ns; E-field 1.1–13.4 MV/m	↑ Ca^2+^ influx with increasing pulse duration (≥11 ns), shift from VGCC-mediated to mixed VGCC + non-VGCC pathways; short pulses (≤5 ns) mimic physiological nAChR activation	Precise tuning of Ca^2+^ influx by ultrashort pulses suggests potential for neuromodulation and electrostimulation therapies without membrane damage	[168]
Gastric adenocarcinoma (EPG85-257P sensitive, EPG85-257RDB resistant)	10 ns, 200 pulses, 12.5–50 kV/cm, 1 kHz	Viability ↓ with dose, Ca^2+^ amplified cytotoxicity; Membrane permeabilization ↑; F-actin reorganization; ROS ↑, GSH/GSSG ↓; Proteasomal activity ↓; Apoptosis ↑ (comet assay, AIF nuclear localization)	nsPEFs + Ca^2+^ overcome drug resistance by inducing oxidative stress, cytoskeletal disruption, proteasomal inhibition, and apoptosis	[9]
Melanoma (A375, C32) & Colon cancer (LoVo, LoVoDX)	10–300 ns, 100–400 pulses, 5–10 kV/cm	Viability ↓ (dose- and Ca^2+^-dependent), Membrane permeabilization ↑, ROS ↑, GSH ↓, Apoptosis ↑, Necrosis ↑ at higher doses, Ca^2+^ influx sustained	nsPEFs + Ca^2+^ induce strong cytotoxicity and oxidative stress, effective against resistant LoVoDX cells; supports Ca-electrochemotherapy development	[169]

## Data Availability

Data are contained within the article.

## Abbreviations

The following abbreviations are used in this manuscript:

nsPEF	Nanosecond pulsed electric field
ICD	Immunogenic cell death
DAMPs	Danger-associated molecular patterns
CSC	Cancer stem cell
nsEP	Nanosecond pulse electroporation
MOMP	mitochondrial outer membrane permeabilization
OMM	Outer mitochondrial membrane
ER	Endoplasmic reticulum
UPR	Unfolded protein response
MAMs	mitochondria-associated membranes
TME	Tumor microenvironment
ICIs	Immune checkpoint inhibitors
ROS	Reactive oxygen species
NPs	Nanoparticles
DOX	Doxorubicin
AI	Artificial intelligence
ATP	Adenosine triphosphate

## References

[1] Beebe S.J., Sain N.M., Ren W. (2013). Induction of Cell Death Mechanisms and Apoptosis by Nanosecond Pulsed Electric Fields (NsPEFs). Cells.

[2] Butkus P., Murauskas A., Tolvaišienė S., Novickij V. (2020). Concepts and Capabilities of In-House Built Nanosecond Pulsed Electric Field (NsPEF) Generators for Electroporation: State of Art. Appl. Sci..

[3] Gianulis E.C., Labib C., Saulis G., Novickij V., Pakhomova O.N., Pakhomov A.G. (2017). Selective Susceptibility to Nanosecond Pulsed Electric Field (NsPEF) across Different Human Cell Types. Cell. Mol. Life Sci..

[4] Beebe S.J., Fox P.M., Rec L.J., Somers K., Stark R.H., Schoenbach K.H. (2002). Nanosecond Pulsed Electric Field (NsPEF) Effects on Cells and Tissues: Apoptosis Induction and Tumor Growth Inhibition. IEEE Trans. Plasma Sci..

[5] Ruiz-Fernández A.R., Campos L., Gutierrez-Maldonado S.E., Núñez G., Villanelo F., Perez-Acle T. (2022). Nanosecond Pulsed Electric Field (NsPEF): Opening the Biotechnological Pandora’s Box. Int. J. Mol. Sci..

[6] Neu W.K., Neu J.C., Efimov I.R., Kroll M.W., Tchou P.J. (2009). Theory of Electroporation BT-Cardiac Bioelectric Therapy: Mechanisms and Practical Implications.

[7] Yin M., Xiao J., Huang G., Xie H., Liu H., Yuan J., Liu X., Chiarini A., Armato U., Prà I.D. (2025). Nanosecond Pulsed Electric Field Applications Rejuvenate Aging Endothelial Cells by Rescuing Mitochondrial-to-Nuclear Retrograde Communication. Regen. Ther..

[8] Hirata M., Tanioka S., Hamada Y., Oyadomari S., Shimomura N. Study on Selection of Appropriate Conditions of Nanosecond Pulsed Electric Field for Activation of Unfolded Protein Response Using GFP-Expressing Cells. Proceedings of the 2024 IEEE International Power Modulator and High Voltage Conference (IPMHVC).

[9] Kulbacka J., Rembiałkowska N., Szewczyk A., Rossowska J., Drąg-Zalesińska M., Kulbacki M., Choromańska A. (2022). Nanosecond PEF Induces Oxidative Stress and Apoptosis via Proteasomal Activity Inhibition in Gastric Adenocarcinoma Cells with Drug Resistance. Int. J. Mol. Sci..

[10] Ford W.E., Ren W., Blackmore P.F., Schoenbach K.H., Beebe S.J. (2010). Nanosecond Pulsed Electric Fields Stimulate Apoptosis without Release of Pro-Apoptotic Factors from Mitochondria in B16f10 Melanoma. Arch. Biochem. Biophys..

[11] Awasthi K., Wu T.-E., Hsu H.-Y., Ohta N. (2023). Application of Nanosecond Pulsed Electric Field and Autofluorescence Lifetime Microscopy of FAD in Lung Cells. J. Phys. Chem. B.

[12] Taibi A., Perrin M.-L., Albouys J., Jacques J., Yardin C., Durand-Fontanier S., Bardet S.M. (2021). 10 Ns PEFs Induce a Histological Response Linked to Cell Death and Cytotoxic T-Lymphocytes in an Immunocompetent Mouse Model of Peritoneal Metastasis. Clin. Transl. Oncol..

[13] Sowa P.W., Novickij V., Kiełbik A., Kollotzek F., Heinzmann D., Borst O., Gawaz M.P. (2025). Fractionation of Nanosecond Pulsed Electric Fields Lowers Lethal Dose by Enhancing Cardiomyocyte Membrane Permeability. Hear. Rhythm.

[14] Liu J., Chen X., Zheng S. (2021). Immune Response Triggered by the Ablation of Hepatocellular Carcinoma with Nanosecond Pulsed Electric Field. Front. Med..

[15] Ruiz-Fernández A.R., Rosemblatt M., Perez-Acle T. (2022). Nanosecond Pulsed Electric Field (NsPEF) and Vaccines: A Novel Technique for the Inactivation of SARS-CoV-2 and Other Viruses?. Ann. Med..

[16] Rossi A., Pakhomova O.N., Pakhomov A.G., Weygandt S., Bulysheva A.A., Murray L.E., Mollica P.A., Muratori C. (2019). Mechanisms and Immunogenicity of NsPEF-Induced Cell Death in B16F10 Melanoma Tumors. Sci. Rep..

[17] Muratori C., Pakhomov A.G., Gianulis E., Meads J., Casciola M., Mollica P.A., Pakhomova O.N. (2017). Activation of the Phospholipid Scramblase TMEM16F by Nanosecond Pulsed Electric Fields (NsPEF) Facilitates Its Diverse Cytophysiological Effects. J. Biol. Chem..

[18] Zhou H., Wang Z., Dong Y., Alhaskawi A., Tu T., Hasan Abdullah Ezzi S., Goutham Kota V., Hasan Abdulla Hasan Abdulla M., Li P., Wu B. (2023). New Advances in Treatment of Skin Malignant Tumors with Nanosecond Pulsed Electric Field: A Literature Review. Bioelectrochemistry.

[19] Beebe S.J., Schoenbach K.H. (2005). Nanosecond Pulsed Electric Fields: A New Stimulus to Activate Intracellular Signaling. BioMed Res. Int..

[20] Huang H., Huang F., Liang X., Fu Y., Cheng Z., Huang Y., Chen Z., Duan Y., Chen Y. (2023). Afatinib Reverses EMT via Inhibiting CD44-Stat3 Axis to Promote Radiosensitivity in Nasopharyngeal Carcinoma. Pharmaceuticals.

[21] Oshin E.A., Minhas Z., Biancatelli R.M.L.C., Catravas J.D., Heller R., Guo S., Jiang C. (2024). Synergistic Effects of Nanosecond Pulsed Plasma and Electric Field on Inactivation of Pancreatic Cancer Cells in Vitro. Sci. Rep..

[22] Casciati A., Taddei A.R., Rampazzo E., Persano L., Viola G., Cani A., Bresolin S., Cesi V., Antonelli F., Mancuso M. (2024). Involvement of Mitochondria in the Selective Response to Microsecond Pulsed Electric Fields on Healthy and Cancer Stem Cells in the Brain. Int. J. Mol. Sci..

[23] Orlacchio R., Kolosnjaj-Tabi J., Mattei N., Lévêque P., Rols M.P., Arnaud-Cormos D., Golzio M. (2023). Effects of Nanosecond Pulsed Electric Field (NsPEF) on a Multicellular Spheroid Tumor Model: Influence of Pulse Duration, Pulse Repetition Rate, Absorbed Energy, and Temperature. Int. J. Mol. Sci..

[24] Li K., Fan L., Lin J., Heng B.C., Deng Z., Zheng Q., Zhang J., Jiang Y., Ge Z. (2022). Nanosecond Pulsed Electric Fields Prime Mesenchymal Stem Cells to Peptide Ghrelin and Enhance Chondrogenesis and Osteochondral Defect Repair in Vivo. Sci. China Life Sci..

[25] Batista Napotnik T., Reberšek M., Vernier P.T., Mali B., Miklavčič D. (2016). Effects of High Voltage Nanosecond Electric Pulses on Eukaryotic Cells (in Vitro): A Systematic Review. Bioelectrochemistry.

[26] Yang H., Zhou H., Fu M., Xu H., Huang H., Zhong M., Zhang M., Hua W., Lv K., Zhu G. (2024). TMEM64 Aggravates the Malignant Phenotype of Glioma by Activating the Wnt/β-Catenin Signaling Pathway. Int. J. Biol. Macromol..

[27] Wu X., Fu M., Ge C., Zhou H., Huang H., Zhong M., Zhang M., Xu H., Zhu G., Hua W. (2024). M6A-Mediated Upregulation of LncRNA CHASERR Promotes the Progression of Glioma by Modulating the MiR-6893-3p/TRIM14 Axis. Mol. Neurobiol..

[28] Fukuda H., Miyake M., Hirai H., Teranishi K., Shimomura N., Oyadomari S. Effects on Endoplasmic Reticulum Stress Response of Applying Nanosecond Pulsed Electric Fields. Proceedings of the 2015 IEEE Pulsed Power Conference (PPC).

[29] Rossi A., Pakhomova N.O., Mollica P.A., Casciola M., Mangalanathan U., Pakhomov G.A., Muratori C. (2019). Nanosecond Pulsed Electric Fields Induce Endoplasmic Reticulum Stress Accompanied by Immunogenic Cell Death in Murine Models of Lymphoma and Colorectal Cancer. Cancers.

[30] Rana J.N., Mumtaz S., Choi E.H., Han I. (2023). ROS Production in Response to High-Power Microwave Pulses Induces P53 Activation and DNA Damage in Brain Cells: Radiosensitivity and Biological Dosimetry Evaluation. Front. Cell Dev. Biol..

[31] Rana J.N., Mumtaz S., Han I., Choi E.H. (2024). Formation of Reactive Species via High Power Microwave Induced DNA Damage and Promoted Intrinsic Pathway-Mediated Apoptosis in Lung Cancer Cells: An in Vitro Investigation. Fundam. Res..

[32] Baker C., Willis A., Milestone W., Baker M., Garner A.L., Joshi R.P. (2024). Numerical Assessments of Geometry, Proximity and Multi-Electrode Effects on Electroporation in Mitochondria and the Endoplasmic Reticulum to Nanosecond Electric Pulses. Sci. Rep..

[33] Zhuang J., Shi F., Guo J., Shao T., Zhang C. (2023). Biomedical Applications of Pulsed Discharge and Pulsed Electric Field BT-Pulsed Discharge Plasmas: Characteristics and Applications.

[34] Zhang Z., He D., Gong W., Xu Z., Wang H., Li Q. Simulation and Mechanism Study of Cavity Discharge Under Pulsed Electric Field. Proceedings of the 2024 IEEE 5th International Conference on Dielectrics (ICD).

[35] Go E.-J., Yang D., Ryu W., Chon H.J., Kim C., Park K.-S., Kim D.-H., Han D.K., Park W. (2021). Optimal Voltage and Electrical Pulse Conditions for Electrical Ablation to Induce Immunogenic Cell Death (ICD). J. Ind. Eng. Chem..

[36] Wang Z.-B., Zhang X., Fang C., Liu X.-T., Liao Q.-J., Wu N., Wang J. (2024). Immunotherapy and the Ovarian Cancer Microenvironment: Exploring Potential Strategies for Enhanced Treatment Efficacy. Immunology.

[37] Huang L., Tan J., Lin P., Chen Z., Huang Q., Yao H., Jiang L., Long B., Long Y. (2024). Autoimmune Encephalitis Followed by Hemophagocytic Lymph Histiocytosis: A Case Report. Front. Immunol..

[38] Tanori M., Casciati A., Zambotti A., Pinto R., Gianlorenzi I., Pannicelli A., Giardullo P., Benassi B., Marino C., Mancuso M. (2021). Microsecond Pulsed Electric Fields: An Effective Way to Selectively Target and Radiosensitize Medulloblastoma Cancer Stem Cells. Int. J. Radiat. Oncol. Biol. Phys..

[39] Lai J., Wang Z., Zhou H., Li P., Lu H., Tu T. (2023). Low-Intensity Nanosecond Pulsed Electric Field Accelerates Osteogenic Transformation of Human Dermal Fibroblasts by Enhancing Cell Pluripotency. Cell. Reprogram..

[40] Rao X., Chen S., Alfadhl Y., Chen X., Sun L., Yu L., Zhou J. (2022). Pulse Width and Intensity Effects of Pulsed Electric Fields on Cancerous and Normal Skin Cells. Sci. Rep..

[41] Batista Napotnik T., Polajžer T., Miklavčič D. (2021). Cell Death Due to Electroporation–A Review. Bioelectrochemistry.

[42] Campelo S.N., Huang P.-H., Buie C.R., Davalos R.V. (2023). Recent Advancements in Electroporation Technologies: From Bench to Clinic. Annu. Rev. Biomed. Eng..

[43] Choi S.-E., Khoo H., Hur S.C. (2022). Recent Advances in Microscale Electroporation. Chem. Rev..

[44] Qian K., Yao C., Wang Y., Yang Q., Xiang S., Pei Q., Zhu T., Liu H., Dong S. (2025). Potential of Ultrashort Pulsed Electric Fields to Disrupt Dense Structure in Glioma Tumors. IEEE Trans. Biomed. Eng..

[45] Ren W., Beebe S.J. (2011). An Apoptosis Targeted Stimulus with Nanosecond Pulsed Electric Fields (NsPEFs) in E4 Squamous Cell Carcinoma. Apoptosis.

[46] Estlack L.E., Roth C.C., Thompson G.L., Lambert W.A., Ibey B.L. (2014). Nanosecond Pulsed Electric Fields Modulate the Expression of Fas/CD95 Death Receptor Pathway Regulators in U937 and Jurkat Cells. Apoptosis.

[47] Lei Y., Dong S., Liang R., Xiang S., Huang Q., Ma J., Kou H., Yu L., Yao C. (2025). Parallel Resonant Magnetic Field Generator for Biomedical Applications. IEEE Trans. Biomed. Circuits Syst..

[48] Radzevičiūtė E., Malyško-Ptašinskė V., Novickij J., Novickij V., Girkontaitė I. (2022). Transfection by Electroporation of Cancer and Primary Cells Using Nanosecond and Microsecond Electric Fields. Pharmaceutics.

[49] Mi Y., Xu J., Liu Q., Wu X., Zhang Q., Tang J. (2021). Single-Cell Electroporation with High-Frequency Nanosecond Pulse Bursts: Simulation Considering the Irreversible Electroporation Effect and Experimental Validation. Bioelectrochemistry.

[50] Fesmire C.C., Williamson R.H., Petrella R.A., Kaufman J.D., Topasna N., Sano M.B. (2024). Integrated Time Nanosecond Pulse Irreversible Electroporation (INSPIRE): Assessment of Dose, Temperature, and Voltage on Experimental and Clinical Treatment Outcomes. IEEE Trans. Biomed. Eng..

[51] Pan J., Chiang C., Wang X., Bertani P., Ma Y., Cheng J., Talesara V., Lee L.J., Lu W. (2023). Cell Membrane Damage and Cargo Delivery in Nano-Electroporation. Nanoscale.

[52] Sözer E.B., Pakhomov A.G., Semenov I., Casciola M., Kim V., Vernier P.T., Zemlin C.W. (2021). Analysis of Electrostimulation and Electroporation by High Repetition Rate Bursts of Nanosecond Stimuli. Bioelectrochemistry.

[53] Milestone W., Hu Q., Loveless A.M., Garner A.L., Joshi R.P. (2022). Modeling Coupled Single Cell Electroporation and Thermal Effects from Nanosecond Electric Pulse Trains. J. Appl. Phys..

[54] Marszalek P., Liu D.S., Tsong T.Y. (1990). Schwan Equation and Transmembrane Potential Induced by Alternating Electric Field. Biophys. J..

[55] Mercadal B., Vernier P.T., Ivorra A. (2016). Dependence of Electroporation Detection Threshold on Cell Radius: An Explanation to Observations Non Compatible with Schwan’s Equation Model. J. Membr. Biol..

[56] Muratori C., Silkuniene G., Mollica P.A., Pakhomov A.G., Pakhomova O.N. (2021). The Role of ESCRT-III and Annexin V in the Repair of Cell Membrane Permeabilization by the Nanosecond Pulsed Electric Field. Bioelectrochemistry.

[57] Lee D., Naikar J.S., Chan S.S.Y., Meivita M.P., Li L., Tan Y.S., Bajalovic N., Loke D.K. (2022). Ultralong Recovery Time in Nanosecond Electroporation Systems Enabled by Orientational-Disordering Processes. Nanoscale.

[58] Gudvangen E., Kim V., Novickij V., Battista F., Pakhomov A.G. (2022). Electroporation and Cell Killing by Milli- to Nanosecond Pulses and Avoiding Neuromuscular Stimulation in Cancer Ablation. Sci. Rep..

[59] Vižintin A., Marković S., Ščančar J., Miklavčič D. (2021). Electroporation with Nanosecond Pulses and Bleomycin or Cisplatin Results in Efficient Cell Kill and Low Metal Release from Electrodes. Bioelectrochemistry.

[60] Sundararajan R. (2009). Nanosecond Electroporation: Another Look. Mol. Biotechnol..

[61] Reberšek M., Miklavčič D. (2011). Advantages and Disadvantages of Different Concepts of Electroporation Pulse Generation. Automatika.

[62] Pakhomov A.G., Pakhomova O.N. (2020). The Interplay of Excitation and Electroporation in Nanosecond Pulse Stimulation. Bioelectrochemistry.

[63] Zhang R., Wang T., Shen H., Zhou X., Han Q., Li L., Zhang L., Wang C., Dong X. (2025). Tumor Microenvironment-Responsive MnOx-Mesoporous Carbon Nanoparticles for Enhanced Chemodynamic Synergistic Antitumor Therapy. ACS Appl. Nano Mater..

[64] Pakhomov A.G., Grigoryev S., Semenov I., Casciola M., Jiang C., Xiao S. (2018). The Second Phase of Bipolar, Nanosecond-Range Electric Pulses Determines the Electroporation Efficiency. Bioelectrochemistry.

[65] Li J., He H.-G., Guan C., Ding Y., Hu X. (2025). Dynamic Joint Prediction Model of Severe Radiation-Induced Oral Mucositis among Nasopharyngeal Carcinoma: A Prospective Longitudinal Study. Radiother. Oncol..

[66] Chen Y., Deng Y., Li Y., Qin Y., Zhou Z., Yang H., Sun Y. (2024). Oxygen-Independent Radiodynamic Therapy: Radiation-Boosted Chemodynamics for Reprogramming the Tumor Immune Environment and Enhancing Antitumor Immune Response. ACS Appl. Mater. Interfaces.

[67] Feng C., Wang Y., Xu J., Zheng Y., Zhou W., Wang Y., Luo C. (2024). Precisely Tailoring Molecular Structure of Doxorubicin Prodrugs to Enable Stable Nanoassembly, Rapid Activation, and Potent Antitumor Effect. Pharmaceutics.

[68] Beebe S.J., Chen Y.-J., Sain N.M., Schoenbach K.H., Xiao S. (2012). Transient Features in Nanosecond Pulsed Electric Fields Differentially Modulate Mitochondria and Viability. PLoS ONE.

[69] Gao Q., Zhang M., Chen R., Teng P., Dai X., Wu B., Hong L., Ma L., Liu L., Wu S. (2025). Microsecond Pulsed Electric Fields Induce Myocardial Ablation by Secondary Mitochondrial Damage and Cell Death Mechanisms. Sci. Rep..

[70] Shang T., Sun G., Shen S., Zhang Y., Han X., Ding W., Xie K., Hu J., Yang Q., Li J. (2023). Simulation of the Effect of Nanosecond Pulsed Electric Field on Mitochondria. The Proceedings of the 17th Annual Conference of China Electrotechnical Society.

[71] Awasthi K., Huang W.-C., Wei C.-Y., Hsu H.-Y., Ohta N. (2025). Unveiling the Susceptibility of Nanosecond Pulsed Electric Field on Intracellular Function in Breast Cancerous and Normal Cells Using Fluorescence Imaging. Biosens. Bioelectron..

[72] Qian K., Gao S., Jiang Z., Ding Q., Cheng Z. (2024). Recent Advances in Mitochondria-Targeting Theranostic Agents. Exploration.

[73] Asadipour K., Zhou C., Yi V., Beebe S.J., Xiao S. (2023). Ultra-Low Intensity Post-Pulse Affects Cellular Responses Caused by Nanosecond Pulsed Electric Fields. Bioengineering.

[74] Kasprzycka W., Trębińska-Stryjewska A., Lewandowski R.B., Stępińska M., Osuchowska P.N., Dobrzyńska M., Achour Y., Osuchowski Ł.P., Starzyński J., Mierczyk Z. (2021). Nanosecond Pulsed Electric Field Only Transiently Affects the Cellular and Molecular Processes of Leydig Cells. Int. J. Mol. Sci..

[75] Xie Y., Zhang L., Li Y., He D., Zheng L. (2021). Chrysophanol Localizes in Mitochondria to Promote Cell Death through Upregulation of Mitochondrial Cyclophilin D in HepG2 Cells. Chin. Herb. Med..

[76] Zhang R., Shan H., Li Y., Ma Y., Liu S., Liu X., Yang X., Zhang J., Zhang M. (2023). Cyclophilin D Contributes to Airway Epithelial Mitochondrial Damage in Chronic Obstructive Pulmonary Disease. Lung.

[77] He X., Jiang Z., Akakuru O.U., Li J., Wu A. (2021). Nanoscale Covalent Organic Frameworks: From Controlled Synthesis to Cancer Therapy. Chem. Commun..

[78] Wu S.A., Li Z.J., Qi L. (2025). Endoplasmic Reticulum (ER) Protein Degradation by ER-Associated Degradation and ER-Phagy. Trends Cell Biol..

[79] Hirata M., Tanioka S., Hamada Y., Oyadomari S., Shimomura N. Study of Appropriate Condition of Nanosecond Pulsed Electric Fields for Induction of Unfolded Protein Response Using GFP-Expressing Cell. Proceedings of the 2023 IEEE Pulsed Power Conference (PPC).

[80] Yao C., Ma X., Qian K., Wang Y., Dong S. (2023). Simulation and Experimental Study on the Responses of Subcellular Structures in Tumor Cells Induced by 5 Ns Pulsed Electric Fields. Appl. Sci..

[81] Cantu J.C., Tolstykh G.P., Tarango M., Beier H.T., Ibey B.L. (2021). Caveolin-1 Is Involved in Regulating the Biological Response of Cells to Nanosecond Pulsed Electric Fields. J. Membr. Biol..

[82] Chen W.-J., Xiong Z.-A., Zhang M., Yao C.-G., Zhao Z.-Y., Hua Y.-Y., Zhou W. (2013). Picosecond Pulsed Electric Fields Induce Apoptosis in HeLa Cells via the Endoplasmic Reticulum Stress and Caspase-Dependent Signaling Pathways. Int. J. Oncol..

[83] Furumoto Y., Sato D., Teranishi K., Shimomura N., Hamada Y., Miyake M., Oyadomari S. Activation of Endoplasmic Reticulum Stress Response by Applying of Nanosecond Pulsed Electric Fields for Medical Application. Proceedings of the 2018 IEEE International Power Modulator and High Voltage Conference (IPMHVC).

[84] Jing R., Jiang Z., Tang X. (2024). Advances in Millimeter-Wave Treatment and Its Biological Effects Development. Int. J. Mol. Sci..

[85] Ji D., Luo Z., Ovcjak A., Alanazi R., Bao M.-H., Feng Z.-P., Sun H.-S. (2022). Role of TRPM2 in Brain Tumours and Potential as a Drug Target. Acta Pharmacol. Sin..

[86] Wang Y., Peng W., Liu H., Dong S., Zhou Q., Yao C. Augmenting the Electrosensitivity of Tumor Cells by Combining Nanosecond and Microsecond Pulsed Electric Fields. Proceedings of the 2024 3rd International Conference on Health Big Data and Intelligent Healthcare (ICHIH).

[87] Yang Q., Kajimoto S., Kobayashi Y., Hiramatsu H., Nakabayashi T. (2021). Regulation of Cell Volume by Nanosecond Pulsed Electric Fields. J. Phys. Chem. B.

[88] Li K., Ning T., Wang H., Jiang Y., Zhang J., Ge Z. (2020). Nanosecond Pulsed Electric Fields Enhance Mesenchymal Stem Cells Differentiation via DNMT1-Regulated OCT4/NANOG Gene Expression. Stem Cell Res. Ther..

[89] Chang A.-Y., Liu X., Tian H., Hua L., Yang Z., Wang S. (2020). Microfluidic Electroporation Coupling Pulses of Nanoseconds and Milliseconds to Facilitate Rapid Uptake and Enhanced Expression of DNA in Cell Therapy. Sci. Rep..

[90] Wang Y., Xu Y., Song J., Liu X., Liu S., Yang N., Wang L., Liu Y., Zhao Y., Zhou W. (2024). Tumor Cell-Targeting and Tumor Microenvironment–Responsive Nanoplatforms for the Multimodal Imaging-Guided Photodynamic/Photothermal/Chemodynamic Treatment of Cervical Cancer. Int. J. Nanomed..

[91] Zhao Y. (2025). Enhancing Panvascular Medicine: Unveiling the Nexus of Pan-Cardio-Oncology and Expanding Therapeutic Frontiers. Sci. Bull..

[92] Hamada Y., Furumoto Y., Izutani A., Taniuchi S., Miyake M., Oyadomari M., Teranishi K., Shimomura N., Oyadomari S. (2020). Nanosecond Pulsed Electric Fields Induce the Integrated Stress Response via Reactive Oxygen Species-Mediated Heme-Regulated Inhibitor (HRI) Activation. PLoS ONE.

[93] Rajabi F., Gusbeth C., Frey W., Maisch J., Nick P. (2020). Nanosecond Pulsed Electrical Fields Enhance Product Recovery in Plant Cell Fermentation. Protoplasma.

[94] Chafai D.E., Vostárek F., Dráberová E., Havelka D., Arnaud-Cormos D., Leveque P., Janáček J., Kubínová L., Cifra M., Dráber P. (2020). Microtubule Cytoskeleton Remodeling by Nanosecond Pulsed Electric Fields. Adv. Biosyst..

[95] You W., Zhou Z., Li Z., Yan J., Wang Y. (2025). From Foe to Friend: Rewiring Oncogenic Pathways through Artificial Selenoprotein to Combat Immune-Resistant Tumor. J. Pharm. Anal..

[96] Du J., Meng X., Yang M., Chen G., Li J., Zhu Z., Wu X., Hu W., Tian M., Li T. (2025). NGR-Modified CAF-Derived Exos Targeting Tumor Vasculature to Induce Ferroptosis and Overcome Chemoresistance in Osteosarcoma. Adv. Sci..

[97] Ninagawa Y., Sugiura R., Kato E., Wada K., Yagi I., Uchida S. (2024). Effects of Pulse Width and Electrical Energy of Low-Voltage Nanosecond Pulsed Electric Fields on Mitochondria in Cancer Cells. IEEJ Trans. Electr. Electron. Eng..

[98] Camera F., Colantoni E., Garcia-Sanchez T., Benassi B., Consales C., Muscat A., Vallet L., Mir L.M., Andre F., Merla C. (2023). In Vitro Imaging and Molecular Characterization of Ca^2+^ Flux Modulation by Nanosecond Pulsed Electric Fields. Int. J. Mol. Sci..

[99] Awasthi K., Chang F.-L., Wu T.-E., Hsu H.-Y., Ohta N. (2022). Modulation of Calcium Signaling by Nanosecond Electric Pulses and Cell Death through Apoptosis in A549 Lung Cancerous Cells. Sens. Actuators B Chem..

[100] Ruiz-Fernández A.R., Campos L., Villanelo F., Gutiérrez-Maldonado S.E., Perez-Acle T. (2021). Exploring the Conformational Changes Induced by Nanosecond Pulsed Electric Fields on the Voltage Sensing Domain of a Ca^2+^ Channel. Membranes.

[101] Joshi R., Beebe S.J., Joshi R., Schoenbach K.H., Xiao S. (2021). Intra-Cellular Calcium Release Dynamics Due to Nanosecond Electric Pulsing BT-Ultrashort Electric Pulse Effects in Biology and Medicine.

[102] Liu Z., Zou Y., Sun Y., Chen X., Chen X., Ren Z. (2021). Effects of Nanosecond Pulsed Electric Fields in Cell Vitality, Apoptosis, and Proliferation of TPC-1 Cells. Anal. Cell. Pathol..

[103] Kim V., Gudvangen E., Kondratiev O., Redondo L., Xiao S., Pakhomov A.G. (2021). Peculiarities of Neurostimulation by Intense Nanosecond Pulsed Electric Fields: How to Avoid Firing in Peripheral Nerve Fibers. Int. J. Mol. Sci..

[104] Haberkorn I., Siegenthaler L., Buchmann L., Neutsch L., Mathys A. (2021). Enhancing Single-Cell Bioconversion Efficiency by Harnessing Nanosecond Pulsed Electric Field Processing. Biotechnol. Adv..

[105] Adamovich I.V., Butterworth T., Orriere T., Pai D.Z., Lacoste D.A., Cha M.S. (2020). Nanosecond Second Harmonic Generation for Electric Field Measurements with Temporal Resolution Shorter than Laser Pulse Duration. J. Phys. D Appl. Phys..

[106] Hruza G.J., Zelickson B.D., Selim M.M., Rohrer T.E., Newman J., Park H., Jauregui L., Nuccitelli R., Knape W.A., Ebbers E. (2020). Safety and Efficacy of Nanosecond Pulsed Electric Field Treatment of Seborrheic Keratoses. Dermatol. Surg..

[107] Asadipour K., Hani M.B., Potter L., Ruedlinger B.L., Lai N., Beebe S.J. (2024). Nanosecond Pulsed Electric Fields (NsPEFs) Modulate Electron Transport in the Plasma Membrane and the Mitochondria. Bioelectrochemistry.

[108] Nanajian A., Scott M., Burcus N.I., Ruedlinger B.L., Oshin E.A., Beebe S.J., Guo S. (2024). Nano-Pulse Treatment Overcomes the Immunosuppressive Tumor Microenvironment to Elicit In Situ Vaccination Protection against Breast Cancer. Vaccines.

[109] Nies M., Watanabe K., Kawamura I., Wang B.J., Litt J., Turovskiy R., Danitz D.J., Uecker D.R., Linder K.E., Maejima Y. (2024). Ablating Myocardium Using Nanosecond Pulsed Electric Fields: Preclinical Assessment of Feasibility, Safety, and Durability. Circ. Arrhythmia Electrophysiol..

[110] Chittams-Miles A.E., Areej M., Purcell E.B., Claudia M. (2023). Nanosecond Pulsed Electric Fields Increase Antibiotic Susceptibility in Methicillin-Resistant Staphylococcus Aureus. Microbiol. Spectr..

[111] Florkowski M., Kuniewski M. (2023). Effects of Nanosecond Impulse and Step Excitation in Pulsed Electro Acoustic Measurements on Signals for Space Charge Determination in High-Voltage Electrical Insulation. Measurement.

[112] Alderman D., Tremble C., Singleton D., Sanders J., Jiang C. (2021). Effects of Pulse Rise Time and Repetition Frequency on Nanosecond Pulsed Plasma Ignition for Combustion. Plasma Res. Express.

[113] Liu H., Shi F., Tang X., Zheng S., Kolb J., Yao C. (2020). Application of Bioimpedance Spectroscopy to Characterize Chemoresistant Tumor Cell Selectivity of Nanosecond Pulse Stimulation. Bioelectrochemistry.

[114] Liu H., Yao C., Zhao Y., Chen X., Dong S., Wang L., Davalos R.V. (2021). In Vitro Experimental and Numerical Studies on the Preferential Ablation of Chemo-Resistant Tumor Cells Induced by High-Voltage Nanosecond Pulsed Electric Fields. IEEE Trans. Biomed. Eng..

[115] Wang Y., Jiang T., Xie L., Wang H., Zhao J., Xu L., Fang C. (2022). Effect of Pulsed Field Ablation on Solid Tumor Cells and Microenvironment. Front. Oncol..

[116] Qian J., Chen T., Wu Q., Zhou L., Zhou W., Wu L., Wang S., Lu J., Wang W., Li D. (2020). Blocking Exposed PD-L1 Elicited by Nanosecond Pulsed Electric Field Reverses Dysfunction of CD8^+^ T Cells in Liver Cancer. Cancer Lett..

[117] Zhao J., Xu M., Sun R., Zhao J., Zhao Q., Wang Y., Tian G., Jiang T. (2022). Single-Cell Analysis Reveals Nanosecond Pulsed Electric Field Ablation Induced Myeloid Cells Remodeling in Pancreatic Cancer. Bioelectrochemistry.

[118] Hu S., Zhu Y., Cheng P., Dai G., Jin L., Wang Q., Chen X., Cai C. (2025). Anticancer Therapy Using FePt Nanoparticles Combined with Nanosecond Pulsed Electric Fields. ACS Appl. Nano Mater..

[119] Zou Y., Sun Y., Chen X., Hong L., Dong G., Bai X., Wang H., Rao B., Ren Z., Yu Z. (2023). Nanosecond Pulse Effectively Ablated Hepatocellular Carcinoma with Alterations in the Gut Microbiome and Serum Metabolites. Front. Pharmacol..

[120] Nuccitelli R., McDaniel A. (2024). Nano-Pulse Stimulation Therapy in Oncology. Bioelectricity.

[121] Ibrahimi N., Vallet L., Andre F.M., Rivaletto M., Novac B.M., Mir L.M., Pécastaing L. (2023). An Overview of Subnanosecond Pulsed Electric Field Biological Effects: Toward Contactless Technologies for Cancer Treatment. Bioelectricity.

[122] Xu Z., Pan C., Chen L., Qian J., Chen X., Zhou L., Zheng S. (2023). Nanosecond Pulsed Electric Field Induces an Antitumor Effect in Triple-Negative Breast Cancer via CXCL9 Axis Dependence in Mice. Cancers.

[123] Ye Z., Zhao Y., Fang F., Song J., Chen H., Xu Y., Wang Z., Li F. (2025). Reactive Oxygen and Nitrogen Species Release of Single Pancreatic Cancer Cells Subjected to Pulsed Electric Field Ablation: Concentration and Dynamics. Anal. Chem..

[124] Sauer N., Szlasa W., Szewczyk A., Novickij V., Saczko J., Baczyńska D., Daczewska M., Kulbacka J. (2023). Effects of Nanosecond Pulsed Electric Field on Immune Checkpoint Receptors in Melanoma Cells. Pharmaceuticals.

[125] Liu Q., Dong W., Liu R., Xu L., Ran L., Xie Z., Lei S., Su X., Yue Z., Xiong D. (2025). Chromatin Landscape Alteration Uncovers Multiple Transcriptional Circuits during Memory CD8^+^ T-Cell Differentiation. Protein Cell.

[126] Liu Z., Li X., Gao Y., Liu J., Feng Y., Liu Y., Wang J., Wang C., Wang D., He J. (2023). Epigenetic Reprogramming of Runx3 Reinforces CD8^+^ T-Cell Function and Improves the Clinical Response to Immunotherapy. Mol. Cancer.

[127] Luo Z., Guo F., Xiang S., Dong S., Yao C., Liu H. (2025). Nanosecond Pulsed Bipolar Cancellation of the Killing Effect on Glioblastoma. IEEE Trans. Biomed. Eng..

[128] Xu M., Zhang W., Xu D., Dong G., Ren Z., Aji T., Ji J., Zhao Q., Pan J., Chen X. (2025). Nanosecond Pulsed Electric Field Ablation as First-Line Curative Therapy for Hepatocellular Carcinoma in High-Risk Locations a Prospective Multicenter. Int. J. Surg..

[129] He L., Yang H., Tang J., Liu Z., Chen Y., Lu B., He H., Tang S., Sun Y., Liu F. (2019). Intestinal Probiotics *E. Coli* Nissle 1917 as a Targeted Vehicle for Delivery of P53 and Tum-5 to Solid Tumors for Cancer Therapy. J. Biol. Eng..

[130] Ma W., Liu H., Xu F., Xu L., Zhang C., Lou W., Xie L., Jiang T. (2025). High-Voltage Electrical Pulses for Pancreatic Cancer Treatment in the Era of Precision Medicine. Int. J. Surg..

[131] Xu L., Wang E., Kang Y., Fu D., Luo L., Quan Y., Xi Y., Huang J., Cui X., Zeng J. (2025). Schottky Nanodiodes Array Enabled Triboelectric Nanosecond Pulse Generator for Ultralow-Cost Tumor Therapy. Device.

[132] Vallin J.R., Azarin S.M. (2025). Leveraging the Immunological Impacts of Irreversible Electroporation as a New Frontier for Cancer Therapy. Annu. Rev. Chem. Biomol. Eng..

[133] Yousfi N., Merbahi N., Bouajila J., Taillandier P., Debouba M. (2025). Microbial Fermentation Assisted by Pulsed Electric Fields, Magnetic Fields and Cold Atmospheric Plasma: State of the Art. Fermentation.

[134] Varghese F., Philpott J.M., Neuber J.U., Hargrave B., Zemlin C.W. (2023). Surgical Ablation of Cardiac Tissue with Nanosecond Pulsed Electric Fields in Swine. Cardiovasc. Eng. Technol..

[135] He W., Zhu J., Feng Y., Liang F., You K., Chai H., Sui Z., Hao H., Li G., Zhao J. (2024). Neuromorphic-Enabled Video-Activated Cell Sorting. Nat. Commun..

[136] Xiao Y., Yu J., Huang Q., Xiao W. (2025). Immunomodulatory Impacts of Thermal and Pulsed Field Ablation Therapy on Hepatocellular Carcinoma Associated with Viral Hepatitis. Die Radiol..

[137] Steelman Z.A., Coker Z.N., Kiester A., Noojin G., Ibey B.L., Bixler J.N. (2021). Quantitative Phase Microscopy Monitors Subcellular Dynamics in Single Cells Exposed to Nanosecond Pulsed Electric Fields. J. Biophotonics.

[138] Rana J.N., Gul K., Mumtaz S. (2025). Isorhamnetin: Reviewing Recent Developments in Anticancer Mechanisms and Nanoformulation-Driven Delivery. Int. J. Mol. Sci..

[139] Rana J.N., Mumtaz S. (2025). Prunin: An Emerging Anticancer Flavonoid. Int. J. Mol. Sci..

[140] Bystrov D.A., Volegova D.D., Korsakova S.A., Salmina A.B., Yurchenko S.O. (2025). Electric Field-Induced Effects in Eukaryotic Cells: Current Progress and Limitations. Tissue Eng. Part B Rev..

[141] Li Q.-G., Liu Z.-G., Dong G., Sun Y., Zou Y.-W., Chen X.-L., Wu B., Chen X.-H., Ren Z.-G. (2023). Nanosecond Pulsed Electric Field Ablates Rabbit VX2 Liver Tumors in a Non-Thermal Manner. PLoS ONE.

[142] Haberkorn I., Buchmann L., Häusermann I., Mathys A. (2021). Nanosecond Pulsed Electric Field Processing of Microalgae Based Biorefineries Governs Growth Promotion or Selective Inactivation Based on Underlying Microbial Ecosystems. Bioresour. Technol..

[143] Hall E.H., Schoenbach K.H., Beebe S.J. (2005). Nanosecond Pulsed Electric Fields (NsPEF) Induce Direct Electric Field Effects and Biological Effects on Human Colon Carcinoma Cells. DNA Cell Biol..

[144] Stacey M., Fox P., Buescher S., Kolb J. (2011). Nanosecond Pulsed Electric Field Induced Cytoskeleton, Nuclear Membrane and Telomere Damage Adversely Impact Cell Survival. Bioelectrochemistry.

[145] Fan A., Liu G., Wu X. (2024). Nanosecond Pulse Electric Field Treatment Initiates Mitochondrial Apoptosis Pathway by Inducing Mitochondrial Morphological Changes in Myocardial Cells. J. Interv. Card. Electrophysiol..

[146] Liu J., Hong M., Li Y., Chen D., Wu Y., Hu Y. (2022). Programmed Cell Death Tunes Tumor Immunity. Front. Immunol..

[147] Kari S., Subramanian K., Altomonte I.A., Murugesan A., Yli-Harja O., Kandhavelu M. (2022). Programmed Cell Death Detection Methods: A Systematic Review and a Categorical Comparison. Apoptosis.

[148] Szlasa W., Michel O., Sauer N., Novickij V., Lewandowski D., Kasperkiewicz P., Tarek M., Saczko J., Kulbacka J. (2023). Nanosecond Pulsed Electric Field Suppresses Growth and Reduces Multi-Drug Resistance Effect in Pancreatic Cancer. Sci. Rep..

[149] Zhang J., Ghasemi N., Zare F., Duley J.A., Cowley D.M., Shaw P.N., Koorts P., Bansal N. (2023). Nanosecond Pulsed Electric Field Treatment of Human Milk: Effects on Microbiological Inactivation, Whey Proteome and Bioactive Protein. Food Chem..

[150] Kulbacka J., Rembiałkowska N., Szewczyk A., Moreira H., Szyjka A., Girkontaitė I., Grela K.P., Novickij V. (2021). The Impact of Extracellular Ca^2+^ and Nanosecond Electric Pulses on Sensitive and Drug-Resistant Human Breast and Colon Cancer Cells. Cancers.

[151] Akter K., Gul K., Mumtaz S. (2025). Revisiting Curcumin in Cancer Therapy: Recent Insights into Molecular Mechanisms, Nanoformulations, and Synergistic Combinations. Curr. Issues Mol. Biol..

[152] Beebe S.J. (2015). Considering Effects of Nanosecond Pulsed Electric Fields on Proteins. Bioelectrochemistry.

[153] Liang Y., Lu Z., Liu H., Huang Q., Zheng X., Li X., Zhou Y. (2025). Anti-Tumor Effects of Nanosecond Pulsed Electric Fields in a Murine Model of Pancreatic Cancer. Bioelectrochemistry.

[154] Lu J.Y., Zhou X., Yang J., Zhou Y., He B., Huang W.T., Wang Y., Guo Z. (2022). Migration Inhibition and Selective Cytotoxicity of Cobalt Hydroxide Nanosheets on Different Cancer Cell Lines. New J. Chem..

[155] Yin S., Chen X., Hu C., Zhang X., Hu Z., Yu J., Feng X., Jiang K., Ye S., Shen K. (2014). Nanosecond Pulsed Electric Field (NsPEF) Treatment for Hepatocellular Carcinoma: A Novel Locoregional Ablation Decreasing Lung Metastasis. Cancer Lett..

[156] Breton M., Mir L.M. (2012). Microsecond and Nanosecond Electric Pulses in Cancer Treatments. Bioelectromagnetics.

[157] Wu S., Wang Y., Guo J., Chen Q., Zhang J., Fang J. (2014). Nanosecond Pulsed Electric Fields as a Novel Drug Free Therapy for Breast Cancer: An in Vivo Study. Cancer Lett..

[158] Liu H., Zhao Y., Yao C., Schmelz E.M., Davalos R.V. (2021). Differential Effects of Nanosecond Pulsed Electric Fields on Cells Representing Progressive Ovarian Cancer. Bioelectrochemistry.

[159] Ren Z., Chen X., Cui G., Yin S., Chen L., Jiang J., Hu Z., Xie H., Zheng S., Zhou L. (2013). Nanosecond Pulsed Electric Field Inhibits Cancer Growth Followed by Alteration in Expressions of NF-ΚB and Wnt/β-Catenin Signaling Molecules. PLoS ONE.

[160] Miao X., Yin S., Shao Z., Zhang Y., Chen X. (2015). Nanosecond Pulsed Electric Field Inhibits Proliferation and Induces Apoptosis in Human Osteosarcoma. J. Orthop. Surg. Res..

[161] Xiang X.-W., Liu H.-T., Liu W., Yan Z.-Y., Zeng Y.-L., Wang Y.-J., Liu J., Chen Y.-C., Yu S.-X., Zhu C.-H. (2023). Revolutionizing Wound Healing: Ultrashort Pulse Electric Fields in Seconds for Highly Aligned Extracellular Matrix and Efficient Cell Migration. Chem. Eng. J..

[162] Tu T., Ouyang C., Li P., Ni Z., Wang Z., Lai J., Chen X., Liu Z., Lu H. (2026). Enhancing Fibroblast-Based Bone Regeneration by Harnessing Nanosecond Pulsed Electric Field. Bioelectrochemistry.

[163] Zheng W., Zhu Y., Chen Q., Shi S., Huang Q., Chen X., Li X., Cheng P., Wu H., Hu S. (2025). Nanosecond Pulsed Electric Field-Empowered Physical–Chemical Cascade Ferroptosis Therapy for Triple-Negative Breast Cancer. J. Mater. Chem. B.

[164] Awasthi K., Li S.-P., Zhu C.-Y., Hsu H.-Y., Ohta N. (2023). Fluorescence Microscopic Approach for Detection of Two Different Modes of Breast Cancer Cell Death Induced by Nanosecond Pulsed Electric Field. Sens. Actuators B Chem..

[165] Qian J., Ding L., Wu Q., Yu X., Li Q., Gu Y., Wang S., Mao J., Liu X., Li B. (2024). Nanosecond Pulsed Electric Field Stimulates CD103^+^ DC Accumulation in Tumor Microenvironment via NK-CD103^+^ DC Crosstalk. Cancer Lett..

[166] Rembiałkowska N., Kucharczyk J., Radzevičiūtė-Valčiukė E., Novickij V., Tonci M., Dündar A., Kulbacka J., Szlasa W. (2024). Enhancing Lung Cancer Growth Inhibition with Calcium Ions: Role of Mid- and High-Frequency Electric Field Pulses. Biomed. Pharmacother..

[167] Liu J., Fang C., Jin X., Tian G., Sun Z., Hong L., Pan J., Chen X., Zhao J., Cao H. (2023). Nanosecond Pulsed Electric Field Ablation-Induced Modulation of Sphingolipid Metabolism Is Associated with Ly6c2^+^ Mononuclear Phagocyte Differentiation in Liver Cancer. Mol. Oncol..

[168] Yun S.H., Mansurov V., Yang L., Yoon J., Leblanc N., Craviso G.L., Zaklit J. (2024). Modulating Ca^2+^ Influx into Adrenal Chromaffin Cells with Short-Duration Nanosecond Electric Pulses. Biophys. J..

[169] Balevičiūtė A., Radzevičiūtė E., Želvys A., Malyško-Ptašinskė V., Novickij J., Zinkevičienė A., Kašėta V., Novickij V., Girkontaitė I. (2022). High-Frequency Nanosecond Bleomycin Electrochemotherapy and Its Effects on Changes in the Immune System and Survival. Cancers.

[170] Franklin S., Beier H.T., Ibey B.L., Nash K. External Stimulation by Nanosecond Pulsed Electric Fields to Enhance Cellular Uptake of Nanoparticles. Proceedings of the SPIE-The International Society for Optical Engineering.

[171] Kulbacka J., Pucek A., Wilk K.A., Dubińska-Magiera M., Rossowska J., Kulbacki M., Kotulska M. (2016). The Effect of Millisecond Pulsed Electric Fields (MsPEF) on Intracellular Drug Transport with Negatively Charged Large Nanocarriers Made of Solid Lipid Nanoparticles (SLN): In Vitro Study. J. Membr. Biol..

[172] Souiade L., Domingo-Diez J., Alcaide C., Gámez B., Gámez L., Ramos M., Serrano Olmedo J.J. (2023). Improving the Efficacy of Magnetic Nanoparticle-Mediated Hyperthermia Using Trapezoidal Pulsed Electromagnetic Fields as an In Vitro Anticancer Treatment in Melanoma and Glioblastoma Multiforme Cell Lines. Int. J. Mol. Sci..

[173] Ahmadi Kamalabadi M., Neshastehriz A., Ghaznavi H., Amini S.M. (2022). Folate Functionalized Gold-Coated Magnetic Nanoparticles Effect in Combined Electroporation and Radiation Treatment of HPV-Positive Oropharyngeal Cancer. Med. Oncol..

[174] Radzevičiūtė-Valčiukė E., Gečaitė J., Želvys A., Zinkevičienė A., Žalnėravičius R., Malyško-Ptašinskė V., Nemeikaitė-Čenienė A., Kašėta V., German N., Novickij J. (2023). Improving NonViral Gene Delivery Using MHz Bursts of Nanosecond Pulses and Gold Nanoparticles for Electric Field Amplification. Pharmaceutics.

[175] Guo F., Xiang J., Zhuo Y., Pei K. (2025). Molecular Dynamics Study of Protein-Mediated Electroporation of Kv Channels Induced by NsPEFs: Advantages of Bipolar Pulses. Biomacromolecules.

[176] Enomoto S., Konishi D., Uto Y., Shimomura N. (2022). Effects of Nanosecond Pulsed Electric Fields Application on Cancer Cell and Combination of Anticancer Drug. Electr. Eng. Jpn..

[177] Ma R., Wang Y., Wang Z., Yin S., Liu Z., Yan K. (2024). Enhanced Cellular Doxorubicin Uptake via Delayed Exposure Following Nanosecond Pulsed Electric Field Treatment: An In Vitro Study. Pharmaceutics.

[178] Rana J.N., Mumtaz S., Han I., Choi E.H. (2024). Harnessing the Synergy of Nanosecond High-Power Microwave Pulses and Cisplatin to Increase the Induction of Apoptosis in Cancer Cells through the Activation of ATR/ATM and Intrinsic Pathways. Free Radic. Biol. Med..

[179] Zhao J., Lu H., Xu D., Sun R., Fang C., Zhao Q., He C., Pan Y., Xu F., Jiang T. (2022). Neutrophil Membrane-Coated Nanoparticles for Enhanced Nanosecond Pulsed Electric Field Treatment of Pancreatic Cancer. Int. J. Hyperth..

[180] Yin S., Chen X., Xie H., Zhou L., Guo D., Xu Y., Wu L., Zheng S. (2016). Nanosecond Pulsed Electric Field (NsPEF) Enhance Cytotoxicity of Cisplatin to Hepatocellular Cells by Microdomain Disruption on Plasma Membrane. Exp. Cell Res..

[181] Novickij V., Malyško V., Želvys A., Balevičiūtė A., Zinkevičienė A., Novickij J., Girkontaitė I. (2020). Electrochemotherapy Using Doxorubicin and Nanosecond Electric Field Pulses: A Pilot in Vivo Study. Molecules.

[182] Rembiałkowska N., Novickij V., Baczyńska D., Dubińska-Magiera M., Saczko J., Rudno-Rudzińska J., Maciejewska M., Kulbacka J. (2022). Micro- and Nanosecond Pulses Used in Doxorubicin Electrochemotherapy in Human Breast and Colon Cancer Cells with Drug Resistance. Molecules.

[183] Lekešytė B., Mickevičiūtė E., Malakauskaitė P., Szewczyk A., Radzevičiūtė-Valčiukė E., Malyško-Ptašinskė V., Želvys A., German N., Ramanavičienė A., Kulbacka J. (2024). Application of Gold Nanoparticles for Improvement of Electroporation-Assisted Drug Delivery and Bleomycin Electrochemotherapy. Pharmaceutics.

[184] Nuccitelli R., Tran K., Sheikh S., Athos B., Kreis M., Nuccitelli P. (2010). Optimized Nanosecond Pulsed Electric Field Therapy Can Cause Murine Malignant Melanomas to Self-Destruct with a Single Treatment. Int. J. Cancer.

[185] de Assis T.A., Dall’Agnol F.F., Forbes R.G. (2022). Field Emitter Electrostatics: A Review with Special Emphasis on Modern High-Precision Finite-Element Modelling. J. Phys. Condens. Matter.

[186] Kulbacka J., Rembiałkowska N., Radzevičiūtė-Valčiukė E., Szewczyk A., Novickij V. (2024). Cardiomyocytes Permeabilization and Electrotransfection by Unipolar and Bipolar Asymmetric Electric Field Pulses. Bioelectricity.

[187] Shu T., Ding L., Fang Z., Yu S., Chen L., Moser M.A.J., Zhang W., Qin Z., Zhang B. (2022). Lethal Electric Field Thresholds for Cerebral Cells With Irreversible Electroporation and H-FIRE Protocols: An In Vitro Three-Dimensional Cell Model Study. J. Biomech. Eng..

[188] Kos B., Mattison L., Ramirez D., Cindrič H., Sigg D.C., Iaizzo P.A., Stewart M.T., Miklavčič D. (2023). Determination of Lethal Electric Field Threshold for Pulsed Field Ablation in Ex Vivo Perfused Porcine and Human Hearts. Front. Cardiovasc. Med..

[189] Lv Y., Liu H., Feng Z., Zhang J., Chen G., Yao C. (2022). The Enlargement of Ablation Area by Electrolytic Irreversible Electroporation (E-IRE) Using Pulsed Field with Bias DC Field. Ann. Biomed. Eng..

[190] Semenov I., Xiao S., Pakhomov A.G. (2013). Primary Pathways of Intracellular Ca^2+^ Mobilization by Nanosecond Pulsed Electric Field. Biochim. Biophys. Acta-Biomembr..

[191] Sanders J.M., Kuthi A., Vernier P.T., Wu Y.-H., Jiang C., Gundersen M.A. Scalable, Compact, Nanosecond Pulse Generator with a High Repetition Rate for Biomedical Applications Requiring Intense Electric Fields. Proceedings of the 2009 IEEE Pulsed Power Conference.

[192] Riaz K., Leung S.-F., Fan Z., Lee Y.-K. (2017). Electric Field Enhanced 3D Scalable Low-Voltage Nano-Spike Electroporation System. Sens. Actuators A Phys..

[193] Vanneman M., Dranoff G. (2012). Combining Immunotherapy and Targeted Therapies in Cancer Treatment. Nat. Rev. Cancer.

[194] Vizintin A., Markovic S., Scancar J., Kladnik J., Turel I., Miklavcic D. (2022). Nanosecond Electric Pulses Are Equally Effective in Electrochemotherapy with Cisplatin as Microsecond Pulses. Radiol. Oncol..

[195] McBride S., Avazzadeh S., Wheatley A.M., O’Brien B., Coffey K., Elahi A., O’Halloran M., Quinlan L.R. (2021). Ablation Modalities for Therapeutic Intervention in Arrhythmia-Related Cardiovascular Disease: Focus on Electroporation. J. Clin. Med..

[196] Rembiałkowska N., Novickij V., Radzevičiūtė-Valčiukė E., Mickevičiūtė E., Gajewska-Naryniecka A., Kulbacka J. (2023). Susceptibility of Various Human Cancer Cell Lines to Nanosecond and Microsecond Range Electrochemotherapy: Feasibility of Multi-Drug Cocktails. Int. J. Pharm..

[197] Yimingjiang M., Tuergan T., Chen X., Wen H., Shao Y., Zhang R., Aihaiti K., Xue J., Aji T., Zhang W. (2020). Comparative Analysis of Immunoactivation by Nanosecond Pulsed Electric Fields and PD-1 Blockade in Murine Hepatocellular Carcinoma. Anal. Cell. Pathol..

